# Supplementary material for: Analyses of hyphal diversity in Trichosporonales yeasts based on fluorescent microscopic observations
Source: Microbiol Spectr. 2025 Feb 25;13(4):e03210-24. doi: 10.1128/spectrum.03210-24 (PMC11960055; doi:10.1128/spectrum.03210-24)
Supplement: Supplemental material — Additional experimental details. [file spectrum.03210-24-s0001.pdf]

Supplemental table 1

Numerical values used for creating graphs are shown. Extended or increased effects are colored by orange. Shorter or decreased effects are colored by blue. Not changed effects are colored by gray. Statistical differences between samples are calculated using Mann-Whitney U Test. The *p*-values are significant at *p* <0.05. Statistically significant values were described in bold.

Fig. 1 and 2

| Strains | | Media | Cell number | Average length ($\mu$m) | Standard deviation ($\mu$m) | Relative value | Statics *p*-value | Impact by magnesium addition |
|---|---|---|---|---|---|---|---|---|
| JCM 2466 | *T. asahii* | Sab | N=401 | 11.9 | 10.5 | 1 | ***p*< 0.00001** | Longer |
| | | Sab+Mg | N=305 | 72.7 | 45.4 | 6.1092437 | | |
| JCM 2937 | *T. asteroides* | Sab | N=293 | 57.3 | 34.7 | 1 | ***p*< 0.00001** | Longer |
| | | Sab+Mg | N=304 | 74.2 | 48.2 | 1.29493892 | | |
| JCM 2938 | *T. coremiiforme* | Sab | N=246 | 46.4 | 31.5 | 1 | ***p*=0.00244** | Longer |
| | | Sab+Mg | N=329 | 64.4 | 54.2 | 1.38793103 | | |
| JCM 2941 | *T. faecale* | Sab | N=307 | 27.9 | 14.9 | 1 | ***p*=0.00188** | Shorter |
| | | Sab+Mg | N=379 | 25.6 | 16.3 | 0.91756272 | | |
| JCM 9195 | *T. inkin* | Sab | N=376 | 10.7 | 5.6 | 1 | ***p*< 0.00001** | Shorter |
| | | Sab+Mg | N=378 | 8.6 | 4 | 0.80373832 | | |
| JCM 9940 | *T. ovoides* | Sab | N=262 | 19.3 | 12.4 | 1 | ***p*< 0.00001** | Longer |
| | | Sab+Mg | N=305 | 48.9 | 40.6 | 2.53367876 | | |
| JCM 3936 | *T. aquatile* | Sab | N=339 | 45.4 | 28.7 | 1 | ***p*< 0.00001** | Longer |
| | | Sab+Mg | N=304 | 90.3 | 46.8 | 1.98898678 | | |
| JCM 8357 | *T. japonicum* | Sab | N=316 | 7.9 | 1.8 | 1 | ***p*=0.00736** | Shorter |
| | | Sab+Mg | N=365 | 7.4 | 1.5 | 0.93670886 | | |
| JCM 1458 | *A. porosum* | Sab | N=383 | 28.46 | 29 | 1 | *p*=0.39532 | Not affected |
| | | Sab+Mg | N=359 | 36.98 | 40.94 | 1.29936753 | | |
| JCM 1599 | *A. brassicae* | Sab | N=322 | 32.33 | 27.59 | 1 | ***p*< 0.00001** | Longer |
| | | Sab+Mg | N=373 | 35.03 | 18.9 | 1.08351376 | | |
| JCM 2947 | *A. laibachii* | Sab | N=283 | 89.82 | 66.24 | 1 | *p*=0.1936 | Not affected |
| | | Sab+Mg | N=319 | 115.27 | 106.89 | 1.28334447 | | |
| JCM 9580 | *A. domesticum* | Sab | N=321 | 14.09 | 6.85 | 1 | ***p*< 0.00001** | Shorter |
| | | Sab+Mg | N=507 | 8.64 | 4.04 | 0.61320085 | | |
| JCM 9937 | *A. montevideense* | Sab | N=362 | 40.66 | 25.43 | 1 | ***p*< 0.00001** | Longer |
| | | Sab+Mg | N=382 | 65.04 | 34.57 | 1.59960649 | | |
| JCM 9941 | *A. gamsii* | Sab | N=298 | 18.35 | 11.76 | 1 | ***p*< 0.00001** | Shorter |
| | | Sab+Mg | N=323 | 15.2 | 14.66 | 0.82833787 | | |
| JCM 10018 | *A. gracile* | Sab | N=305 | 26.43 | 23.89 | 1 | ***p*< 0.00001** | Shorter |
| | | Sab+Mg | N=418 | 12.85 | 9.72 | 0.48618994 | | |
| JCM 10691 | *A. veenhuisii* | Sab | N=419 | 37.59 | 30.43 | 1 | ***p*< 0.00001** | Longer |
| | | Sab+Mg | N=393 | 52.34 | 35.91 | 1.39239159 | | |
| HIS 16 | *C. spelunceum* | Sab | N=402 | 25.38 | 12.23 | 1 | ***p*< 0.00001** | Shorter |
| | | Sab+Mg | N=367 | 15.19 | 6.44 | 0.59850276 | | |
| HIS 19 | *C. cavernicola* | Sab | N=417 | 24.62 | 10.66 | 1 | ***p*< 0.00001** | Longer |
| | | Sab+Mg | N=430 | 30.84 | 15.43 | 1.25264013 | | |
| JCM 1462 | *C. cutaneum* | Sab | N=373 | 45.62 | 30.26 | 1 | ***p*=0.02852** | Longer |
| | | Sab+Mg | N=341 | 53.22 | 36.96 | 1.1665936 | | |
| JCM 1532 | *C. curvatum* | Sab | N=431 | 11.62 | 5.04 | 1 | ***p*< 0.00001** | Shorter |
| | | Sab+Mg | N=514 | 8.77 | 3.19 | 0.75473322 | | |
| JCM 9939 | *C. mucoides* | Sab | N=390 | 16.51 | 6.18 | 1 | ***p*< 0.00001** | Shorter |
| | | Sab+Mg | N=474 | 12.64 | 6.04 | 0.76559661 | | |

| | | | | | | | | |
|---|---|---|---|---|---|---|---|---|
| JCM 11170 | *C. dermatis* | Sab | N=462 | 14.79 | 7.09 | 1 | **$p=0.0455$** | Longer |
| | | Sab+Mg | N=426 | 18.33 | 13.13 | 1.23935091 | | |
| JCM 31833 | *C. cyanovorans* | Sab | N=366 | 7.67 | 2.44 | 1 | **$p< 0.00001$** | Shorter |
| | | Sab+Mg | N=387 | 6.37 | 1.89 | 0.83050847 | | |
| JCM 11166 | *C. daszewskae* | Sab | N=399 | 7.72 | 2.78 | 1 | **$p< 0.00001$** | Shorter |
| | | Sab+Mg | N=452 | 6.68 | 3.03 | 0.86528497 | | |
| JCM 14201 | *C. arboriforme* | Sab | N=522 | 7.19 | 2.55 | 1 | **$p< 0.00001$** | Shorter |
| | | Sab+Mg | N=575 | 6.1 | 2.53 | 0.84840056 | | |
| JCM 12878 | *Ta. koratensis* | Sab | N=549 | 7.07 | 1.98 | 1 | **$p< 0.00001$** | Longer |
| | | Sab+Mg | N=401 | 7.82 | 1.77 | 1.10608204 | | |
| JCM 11965 | *Ta. tepidaria* | Sab | N=396 | 7.79 | 2.83 | 1 | **$p=0.03486$** | Shorter |
| | | Sab+Mg | N=357 | 7.49 | 2.17 | 0.96148909 | | |
| JCM 10690 | *P. guehoae* | Sab | N=393 | 72.12 | 37.82 | 1 | **$p=0.00652$** | Longer |
| | | Sab+Mg | N=345 | 75.79 | 70.42 | 1.05088741 | | |
| JCM 1530 | *Pr. fragicola* | Sab | N=541 | 7.75 | 2.83 | 1 | **$p< 0.00001$** | Shorter |
| | | Sab+Mg | N=524 | 6.29 | 1.93 | 0.8116129 | | |
| JCM 1457 | *V. humicola* | Sab | N=399 | 16.92 | 9.1 | 1 | $p=0.40654$ | Not affected |
| | | Sab+Mg | N=376 | 16.38 | 8.66 | 0.96808511 | | |

Fig. 4

| Strains | | Media | Cell number | No septum | Single septum | Multi-septa | Rate of septum cells (%) | Increase rate of septation (%) | Rate of multi-septa cells (%) | Increase rate of multi-septation (%) | Impact by magnesium addition |
|---|---|---|---|---|---|---|---|---|---|---|---|
| JCM 2466 | *T. asahii* | Sab | 623 | 476 | 99 | 48 | 23.5955056 | 3.94327122 | 32.6530612 | 2.65225857 | Increased |
| | | Sab+Mg | 345 | 24 | 43 | 278 | 93.0434783 | | 86.6043614 | | |
| JCM 2937 | *T. asteroides* | Sab | 231 | 154 | 72 | 5 | 33.3333333 | 1.82113821 | 6.49350649 | 3.50625 | Increased |
| | | Sab+Mg | 369 | 145 | 173 | 51 | 60.704607 | | 22.7678571 | | |
| JCM 2938 | *T. coremiiforme* | Sab | 221 | 73 | 100 | 48 | 66.9683258 | 1.05136514 | 32.4324324 | 2.27898551 | Increased |
| | | Sab+Mg | 196 | 58 | 36 | 102 | 70.4081633 | | 73.9130435 | | |
| JCM 2941 | *T. faecale* | Sab | 205 | 180 | 24 | 1 | 12.195122 | 4.27185629 | 4 | 5.8908046 | Increased |
| | | Sab+Mg | 334 | 160 | 133 | 41 | 52.0958084 | | 23.5632184 | | |
| JCM 9195 | *T. inkin* | Sab | 238 | 193 | 39 | 6 | 18.907563 | 0.90819304 | 13.3333333 | 1.54411765 | Not affected |
| | | Sab+Mg | 198 | 164 | 27 | 7 | 17.1717172 | | 20.5882353 | | |
| JCM 9940 | *T. ovoides* | Sab | 110 | 107 | 3 | 0 | 2.72727273 | 18.9393939 | 0 | 58.4 | Increased |
| | | Sab+Mg | 242 | 117 | 52 | 73 | 51.6528926 | | 58.4 | | |
| JCM 3936 | *T. aquatile* | Sab | 228 | 144 | 59 | 25 | 36.8421053 | 2.58579882 | 29.7619048 | 2.83826087 | Increased |
| | | Sab+Mg | 169 | 8 | 25 | 136 | 95.2662722 | | 84.4720497 | | |
| JCM 8357 | *T. japonicum* | Sab | 287 | 67 | 220 | 0 | 76.6550523 | 0.98335055 | 0 | 0 | Not affected |
| | | Sab+Mg | 264 | 65 | 199 | 0 | 75.3787879 | | 0 | | |
| JCM 1458 | *A. porosum* | Sab | 84 | 68 | 7 | 9 | 19.047619 | 1.39285714 | 56.25 | 0.41025641 | Not affected |
| | | Sab+Mg | 196 | 144 | 40 | 12 | 26.5306122 | | 23.0769231 | | |
| JCM 1599 | *A. brassicae* | Sab | 182 | 177 | 4 | 1 | 2.74725275 | 2.22159624 | 20 | 0.76923077 | Not affected |
| | | Sab+Mg | 213 | 200 | 11 | 2 | 6.10328638 | | 15.3846154 | | |
| JCM 2947 | *A. laibachii* | Sab | 119 | 70 | 34 | 15 | 41.1764706 | 0.89164941 | 30.6122449 | 0.73070175 | Not affected |
| | | Sab+Mg | 207 | 131 | 59 | 17 | 36.7149758 | | 22.3684211 | | |
| JCM 9580 | *A. domesticum* | Sab | 387 | 377 | 10 | 0 | 2.58397933 | 1.86301969 | 0 | 0 | Not affected |
| | | Sab+Mg | 457 | 435 | 22 | 0 | 4.81400438 | | 0 | | |
| JCM 9937 | *A. montevideense* | Sab | 125 | 118 | 7 | 0 | 5.6 | 0.87535014 | 0 | 0 | Not affected |
| | | Sab+Mg | 102 | 97 | 5 | 0 | 4.90196078 | | 0 | | |

| | | | | | | | | | | | |
|---|---|---|---|---|---|---|---|---|---|---|---|
| JCM 9941 | *A. gamsii* | Sab | 363 | 241 | 102 | 20 | 33.6088154 | 0.4366895 | 16.3934426 | 0.93050847 | Not affected |
| | | Sab+Mg | 402 | 343 | 50 | 9 | 14.6766169 | | 15.2542373 | | |
| JCM 10018 | *A. gracile* | Sab | 83 | 80 | 3 | 0 | 3.61445783 | 1.51957071 | 0 | 0 | Not affected |
| | | Sab+Mg | 528 | 499 | 29 | 0 | 5.49242424 | | 0 | | |
| JCM 10691 | *A. veenhuisii* | Sab | 110 | 101 | 8 | 1 | 8.18181818 | 0.8045977 | 11.1111111 | 0.42857143 | Not affected |
| | | Sab+Mg | 319 | 298 | 20 | 1 | 6.5830721 | | 4.76190476 | | |
| HIS 16 | *C. spelunceum* | Sab | 189 | 162 | 27 | 0 | 14.2857143 | 0.58513932 | 0 | 0 | Not affected |
| | | Sab+Mg | 323 | 296 | 27 | 0 | 8.35913313 | | 0 | | |
| HIS19 | *C. cavernicola* | Sab | 148 | 130 | 16 | 2 | 12.1621622 | 0.92989418 | 11.1111111 | 1.42105263 | Not affected |
| | | Sab+Mg | 168 | 149 | 16 | 3 | 11.3095238 | | 15.7894737 | | |
| JCM 1462 | *C. cutaneum* | Sab | 176 | 160 | 15 | 1 | 9.09090909 | 1.55905512 | 6.25 | 0.88888889 | Not affected |
| | | Sab+Mg | 127 | 109 | 17 | 1 | 14.1732283 | | 5.55555556 | | |
| JCM 1532 | *C. curvatus* | Sab | 386 | 338 | 48 | 0 | 12.4352332 | 0.71932339 | 0 | 0 | Not affected |
| | | Sab+Mg | 436 | 397 | 39 | 0 | 8.94495413 | | 0 | | |
| JCM 9939 | *C. mucoides* | Sab | 327 | 305 | 22 | 0 | 6.72782875 | 0.95688646 | 0 | 6.67 | Not affected |
| | | Sab+Mg | 466 | 436 | 28 | 2 | 6.43776824 | | 6.66666667 | | |
| JCM 11170 | *C. dermatis* | Sab | 328 | 275 | 43 | 10 | 16.1585366 | 3.30575413 | 18.8679245 | 1.69476744 | Increased |
| | | Sab+Mg | 322 | 150 | 117 | 55 | 53.4161491 | | 31.9767442 | | |
| JCM 31833 | *C. cyanovorans* | Sab | 400 | 354 | 46 | 0 | 11.5 | 0.96829315 | 0 | 0 | Not affected |
| | | Sab+Mg | 458 | 407 | 51 | 0 | 11.1353712 | | 0 | | |
| JCM 11166 | *C. daszewskae* | Sab | 282 | 264 | 18 | 0 | 6.38297872 | 0.15309446 | 0 | 0 | Not affected |
| | | Sab+Mg | 307 | 304 | 3 | 0 | 0.9771987 | | 0 | | |
| JCM 14201 | *C. arboriforme* | Sab | 318 | 306 | 12 | 0 | 3.77358491 | 0.625 | 0 | 0 | Not affected |
| | | Sab+Mg | 424 | 414 | 10 | 0 | 2.35849057 | | 0 | | |
| JCM 12878 | *T. tepidarius* | Sab | 418 | 399 | 18 | 1 | 4.54545455 | 0.58201058 | 5.26315789 | 1.9 | Not affected |
| | | Sab+Mg | 378 | 368 | 9 | 1 | 2.64550265 | | 10 | | |
| JCM 11965 | *T. koratensis* | Sab | 341 | 321 | 19 | 1 | 5.86510264 | 0.35520833 | 5 | 2.85714286 | Not affected |
| | | Sab+Mg | 336 | 329 | 6 | 1 | 2.08333333 | | 14.2857143 | | |
| JCM 10690 | *P. guehoae* | Sab | 74 | 43 | 28 | 3 | 41.8918919 | 0.32258065 | 9.67741935 | 1.03333333 | Not affected |
| | | Sab+Mg | 74 | 64 | 9 | 1 | 13.5135135 | | 10 | | |
| JCM 1530 | *P. fragicola* | Sab | 274 | 254 | 19 | 1 | 7.29927007 | 1.61948882 | 5 | 0 | Not affected |
| | | Sab+Mg | 313 | 276 | 37 | 0 | 11.8210863 | | 0 | | |
| JCM 1457 | *V. humicola* | Sab | 278 | 261 | 17 | 0 | 6.11510791 | 1.00412797 | 0 | 0 | Not affected |
| | | Sab+Mg | 342 | 321 | 21 | 0 | 6.14035088 | | 0 | | |

Fig. 5

| Strains | | Media | Cell number | Average cellular area ($\mu m^2$) | Average number of LDs | LD number per unit area | Ratio | Statics *p*-value | Impact by magnesium addition |
|---|---|---|---|---|---|---|---|---|---|
| JCM 2466 | *T. asahii* | Sab | 16 | 101.2 | 9.6875 | 0.09571187 | 1 | *p*=0.98404 | Not affected |
| | | Sab+Mg | 14 | 233.3 | 23.2142857 | 0.09950484 | 1.03962904 | | |
| JCM 2937 | *T. asteroides* | Sab | 15 | 106.29 | 17.4 | 0.16370205 | 1 | *p*=0.98404 | Not affected |
| | | Sab+Mg | 18 | 109.87 | 17.0555556 | 0.15523289 | 0.94826479 | | |
| JCM 2938 | *T. coremiiforme* | Sab | 12 | 230.02 | 24.5 | 0.1065121 | 1 | *p*=0.12602 | Not affected |
| | | Sab+Mg | 12 | 380.19 | 29.5833333 | 0.07781215 | 0.73054752 | | |
| JCM 2941 | *T. faecale* | Sab | 12 | 88.24 | 9.83333333 | 0.11143508 | 1 | ***p*=0.00512** | decreased |
| | | Sab+Mg | 12 | 83.3 | 5.25 | 0.06302554 | 0.56558078 | | |
| JCM 9195 | *T. inkin* | Sab | 13 | 30.7 | 5.76923077 | 0.18792004 | 1 | ***p*=0.04136** | decreased |
| | | Sab+Mg | 12 | 31.14 | 4 | 0.1284599 | 0.68358808 | | |
| JCM 9940 | *T. ovoides* | Sab | 12 | 81.06 | 11 | 0.13570895 | 1 | *p*=0.93624 | Not affected |
| | | Sab+Mg | 13 | 144.7 | 20.5384615 | 0.14194027 | 1.04591679 | | |

| | | | | | | | | | |
|---|---|---|---|---|---|---|---|---|---|
| JCM 3936 | *T. aquatile* | Sab | 12 | 193.01 | 24.75 | 0.1282345 | 1 | **_p_=0.00038** | Increased |
| | | Sab+Mg | 12 | 338.22 | 62.3333333 | 0.18429774 | 1.43719311 | | |
| JCM 8357 | *T. japonicum* | Sab | 21 | 39.67 | 6.0952381 | 0.15362943 | 1 | *p*=0.28014 | Not affected |
| | | Sab+Mg | 10 | 49.1 | 5.9 | 0.12016783 | 0.78219277 | | |
| JCM 1458 | *A. porosum* | Sab | 14 | 193.71 | 27.5 | 0.14196406 | 1 | *p*=0.1902 | Not affected |
| | | Sab+Mg | 12 | 207.09 | 23.25 | 0.11227058 | 0.79083805 | | |
| JCM 1599 | *A. brassicae* | Sab | 14 | 91.12 | 15.3571429 | 0.16853928 | 1 | **_p_=0.00578** | Increased |
| | | Sab+Mg | 17 | 128.87 | 30.4705882 | 0.23645178 | 1.40294761 | | |
| JCM 2947 | *A. laibachii* | Sab | 14 | 361.01 | 35.5714286 | 0.0985334 | 1 | *p*=0.06148 | Not affected |
| | | Sab+Mg | 13 | 366.71 | 45.1538462 | 0.12313119 | 1.24963911 | | |
| JCM 9580 | *A. domesticum* | Sab | 20 | 62.36 | 10.35 | 0.16597794 | 1 | **_p_=0.0005** | Increased |
| | | Sab+Mg | 20 | 31.33 | 8.05 | 0.2569024 | 1.54781051 | | |
| JCM 9937 | *A. montevideense* | Sab | 15 | 127.22 | 14.3333333 | 0.11266314 | 1 | **_p_=0.00006** | Increased |
| | | Sab+Mg | 15 | 136.63 | 31.0666667 | 0.22738111 | 2.01823871 | | |
| JCM 9941 | *A. gamsii* | Sab | 15 | 70.11 | 5.53333333 | 0.07892398 | 1 | **_p_< 0.00001** | Increased |
| | | Sab+Mg | 15 | 62.06 | 13 | 0.2094829 | 2.65423639 | | |
| JCM 10018 | *A. gracile* | Sab | 15 | 87.43 | 9.66666667 | 0.11056428 | 1 | **_p_=0.02034** | Increased |
| | | Sab+Mg | 15 | 58.13 | 9.33333333 | 0.160564 | 1.45222309 | | |
| JCM 10691 | *A. veenhuisii* | Sab | 15 | 222.86 | 20.9333333 | 0.09393182 | 1 | *p*=0.77182 | Not affected |
| | | Sab+Mg | 15 | 353.92 | 26.6 | 0.07919502 | 0.84311174 | | |
| HIS 16 | *C. spelunceum* | Sab | 19 | 70.33 | 12.3157895 | 0.17511431 | 1 | **_p_=0.00034** | Increased |
| | | Sab+Mg | 20 | 48.47 | 12.95 | 0.26719037 | 1.52580546 | | |
| HIS 19 | *C. cavernicola* | Sab | 15 | 93.68 | 22.8 | 0.24338173 | 1 | **_p_=0.03078** | decreased |
| | | Sab+Mg | 15 | 154.64 | 29.4 | 0.19011899 | 0.78115555 | | |
| JCM 1462 | *C. cutaneum* | Sab | 15 | 140.34 | 17.3333333 | 0.12350736 | 1 | *p*=0.90448 | Not affected |
| | | Sab+Mg | 15 | 171.32 | 21.1333333 | 0.12335563 | 0.99877149 | | |
| JCM 1532 | *C. curvatum* | Sab | 16 | 37.24 | 3.9375 | 0.10574717 | 1 | **_p_=0.01108** | Increased |
| | | Sab+Mg | 16 | 21.99 | 3.5625 | 0.16201553 | 1.53210275 | | |
| JCM 9939 | *C. mucoides* | Sab | 15 | 59.29 | 6.46666667 | 0.1090674 | 1 | **_p_=0.0466** | decreased |
| | | Sab+Mg | 15 | 56.66 | 4.53333333 | 0.08001231 | 0.73360427 | | |
| JCM 11170 | *C. dermatis* | Sab | 15 | 53.9 | 5.66666667 | 0.10513344 | 1 | **_p_=0.00008** | Increased |
| | | Sab+Mg | 15 | 80.93 | 15 | 0.18534536 | 1.76295344 | | |
| JCM 31833 | *C. cyanovorans* | Sab | 13 | 23.27 | 1.92307692 | 0.08263027 | 1 | **_p_=0.00222** | Increased |
| | | Sab+Mg | 15 | 18.03 | 2.8 | 0.15529708 | 1.87942119 | | |
| JCM 11166 | *C. daszewskae* | Sab | 16 | 41.88 | 13 | 0.31043885 | 1 | **_p_=0.00782** | decreased |
| | | Sab+Mg | 16 | 29.21 | 6.4375 | 0.22040487 | 0.70997837 | | |
| JCM 14201 | *C. arboriforme* | Sab | 21 | 18.69 | 2.85714286 | 0.15288572 | 1 | **_p_=0.00906** | Increased |
| | | Sab+Mg | 22 | 19.14 | 4.59090909 | 0.23990419 | 1.5691733 | | |
| JCM 12878 | *Ta. koratensis* | Sab | 20 | 20.56 | 6.45 | 0.3136473 | 1 | *p*=0.58232 | Not affected |
| | | Sab+Mg | 20 | 18.95 | 5.5 | 0.29025278 | 0.92541138 | | |
| JCM 11965 | *Ta. tepidaria* | Sab | 16 | 21.61 | 4.5 | 0.20827909 | 1 | **_p_< 0.00001** | Increased |
| | | Sab+Mg | 17 | 22.97 | 9.41176471 | 0.40976259 | 1.96737267 | | |
| JCM 10690 | *P. guehoae* | Sab | 12 | 253.11 | 31 | 0.1224776 | 1 | **_p_=0.00124** | decreased |
| | | Sab+Mg | 16 | 247.09 | 11.1875 | 0.04527634 | 0.36967037 | | |
| JCM 1530 | *Pr. fragicola* | Sab | 16 | 29.55 | 4.875 | 0.16497462 | 1 | *p*=0.37346 | Not affected |
| | | Sab+Mg | 16 | 20.72 | 3.0625 | 0.14779514 | 0.89586592 | | |
| JCM 1457 | *V. humicola* | Sab | 16 | 54.58 | 10.6875 | 0.19582598 | 1 | *p*=0.98404 | Not affected |
| | | Sab+Mg | 16 | 49.5 | 9.0625 | 0.18306287 | 0.93482423 | | |

Fig. 6 and 7

| Strains | | Media | Cell number | Vacuole number | Average vacuole area less than 10 $\mu m^2$ | Average vacuole area more than 10 $\mu m^2$ | Average cellular area ($\mu m^2$) | Corrected by cellular area: Average vacuole area less than 10 $\mu m^2$ | Corrected by cellular area: Average vacuole area more than 10 $\mu m^2$ | Total corrected area of vacuoles ($\mu m^2$) | Ratio: Vacuole area less than 10 $\mu m^2$ | Ratio: Vacuole area more than 10 $\mu m^2$ | Total ratios of vacuole area described in Fig. 6 and 7 | Relative values of larger vacuoles | Strains | Statics p-values Upper: relative value of vacuoles ≥10 $\mu m^2$ (*) Lower: total corrected area of vacuoles(**) | Impact by magnesium addition |
|---|---|---|---|---|---|---|---|---|---|---|---|---|---|---|---|---|---|
| JCM 2466 | *T. asahii* | Sab | 16 | 144 | 8.505625 | 3.02625 | 101.2 | 8.505625 | 3.02625 | 11.531875 | 0.7375752 | 0.2624248 | 1 | 0.35579396 | JCM 2466 | ***p*=0.00111** | Increased |
| | | Sab+Mg | 14 | 201 | 32.2835714 | 46.1635714 | 233.3 | 14.0038467 | 20.0246611 | 34.0285077 | 1.21435991 | 1.73646186 | 2.95082176 | 1.42994004 | | ***p*=0.00022** | |
| JCM 2937 | *T. asteroides* | Sab | 15 | 90 | 11.6806667 | 10.9913333 | 106.29 | 11.6806667 | 10.9913333 | 22.672 | 0.51520231 | 0.48479769 | 1 | 0.9409851 | JCM 2937 | *p*=0.11314 | Not affected |
| | | Sab+Mg | 18 | 119 | 11.9844444 | 20.1983333 | 109.87 | 11.5939437 | 19.5401916 | 31.1341353 | 0.51137719 | 0.86186448 | 1.37324168 | 1.6853792 | | *p*=0.10565 | |
| JCM 2938 | *T. coremiiforme* | Sab | 12 | 117 | 26.0633333 | 67.1908333 | 230.02 | 26.0633333 | 67.1908333 | 93.2541666 | 0.27948706 | 0.72051294 | 1 | 2.57798312 | JCM 2938 | *p*=0.46414 | Not affected |
| | | Sab+Mg | 12 | 204 | 44.15 | 107.329167 | 380.19 | 26.7113364 | 64.9355717 | 91.6469081 | 0.28643585 | 0.6963289 | 0.98276475 | 2.43101171 | | *p*=0.48803 | |
| JCM 2941 | *T. faecale* | Sab | 12 | 75 | 16.5883333 | 10.3841667 | 88.24 | 16.5883333 | 10.3841667 | 26.9725 | 0.61500911 | 0.38499089 | 1 | 0.62599217 | JCM 2941 | *p*=0.48803 | Not affected |
| | | Sab+Mg | 12 | 97 | 12.8766667 | 11.9125 | 83.3 | 13.640301 | 12.6189556 | 26.2592565 | 0.50571141 | 0.46784523 | 0.97355664 | 0.92512296 | | *p*=0.35197 | |
| JCM 9195 | *T. inkin* | Sab | 13 | 54 | 2.14461538 | 0 | 30.7 | 2.14461538 | 0 | 2.14461538 | 1 | 0 | 1 | 0 | JCM 9195 | *p*=0.3707 | Increased |
| | | Sab+Mg | 12 | 72 | 10.3941667 | 0.85333333 | 31.14 | 10.2472999 | 0.84127595 | 11.0885758 | 4.77815274 | 0.39227358 | 5.17042632 | 0.08209733 | | ***p*=0.0001** | |
| JCM 9940 | *T. ovoides* | Sab | 12 | 72 | 9.7875 | 6.03166667 | 81.06 | 9.7875 | 6.03166667 | 15.8191667 | 0.61871148 | 0.38128852 | 1 | 0.61626224 | JCM 9940 | *p*=0.38209 | Increased |
| | | Sab+Mg | 13 | 194 | 28.9384615 | 13.3715385 | 144.7 | 16.2111381 | 7.490649 | 23.7017871 | 1.02477826 | 0.47351729 | 1.49829556 | 0.46206805 | | ***p*=0.04363** | |
| JCM 3936 | *T. aquatile* | Sab | 12 | 117 | 21.0733333 | 18.7458333 | 193.01 | 21.0733333 | 18.7458333 | 39.8191666 | 0.52922588 | 0.47077412 | 1 | 0.88955236 | JCM 3936 | *p*=0.119 | Increased |
| | | Sab+Mg | 12 | 223 | 47.5775 | 61.1833333 | 338.22 | 27.1507695 | 34.9151297 | 62.0658992 | 0.68185178 | 0.8768423 | 1.55869408 | 1.28597201 | | ***p*=0.01321** | |
| JCM 8357 | *T. japonicum* | Sab | 21 | 96 | 7.1547619 | 1.31190476 | 39.67 | 7.1547619 | 1.31190476 | 8.46666666 | 0.84505062 | 0.15494938 | 1 | 0.18336106 | JCM 8357 | *p*=0.35942 | Not affected |
| | | Sab+Mg | 10 | 40 | 6.587 | 2.167 | 49.1 | 5.32192037 | 1.75081242 | 7.07273279 | 0.62857327 | 0.20678887 | 0.83536214 | 0.32898133 | | *p*=0.19215 | |
| JCM 1458 | *A. porosum* | Sab | 14 | 103 | 16.5985714 | 51.76 | 193.71 | 16.5985714 | 51.76 | 68.3585714 | 0.24281624 | 0.75718376 | 1 | 3.11834065 | JCM 1458 | *p*=0.31207 | Not affected |
| | | Sab+Mg | 12 | 125 | 19.0916667 | 63.1275 | 207.09 | 17.8581619 | 59.0488581 | 76.90702 | 0.26124247 | 0.86381059 | 1.12505306 | 3.30654735 | | *p*=0.40905 | |
| JCM 1599 | *A. brassicae* | Sab | 14 | 96 | 18.1264286 | 5.89285714 | 91.12 | 18.1264286 | 5.89285714 | 24.0192857 | 0.75466143 | 0.24533857 | 1 | 0.32509753 | JCM 1599 | *p*=0.12924 | Not affected |
| | | Sab+Mg | 17 | 144 | 15.7111765 | 16.5629412 | 128.87 | 11.108888 | 11.7111446 | 22.8200326 | 0.46249868 | 0.48757256 | 0.95007124 | 1.05421393 | | *p*=0.25785 | |
| JCM 2947 | *A. laibachii* | Sab | 14 | 164 | 21.6178571 | 104.335714 | 361.01 | 21.6178571 | 104.335714 | 125.953571 | 0.17163354 | 0.82836646 | 1 | 4.82636709 | JCM 2947 | *p*=0.48006 | Not affected |
| | | Sab+Mg | 13 | 261 | 37.1392308 | 119.052308 | 366.71 | 36.5619528 | 117.201804 | 153.763757 | 0.29028119 | 0.93051593 | 1.22079712 | 3.20556741 | | *p*=0.3409 | |
| JCM 9580 | *A. domesticum* | Sab | 20 | 103 | 11.986 | 4.8485 | 62.36 | 11.986 | 4.8485 | 16.8345 | 0.71199026 | 0.28800974 | 1 | 0.4045136 | JCM 9580 | *p*=0.10383 | decreased |
| | | Sab+Mg | 20 | 106 | 5.4405 | 0.556 | 31.33 | 10.8289046 | 1.10667603 | 11.9355806 | 0.64325668 | 0.06573857 | 0.70899525 | 0.10219649 | | ***p*=0.01463** | |
| JCM 9937 | *A. montevideense* | Sab | 15 | 107 | 16.8446667 | 36.15 | 127.22 | 16.8446667 | 36.15 | 52.9946667 | 0.31785589 | 0.68214411 | 1 | 2.14607986 | JCM 9937 | ***p*=0.03074** | decreased |
| | | Sab+Mg | 15 | 161 | 28.688 | 22.4453333 | 136.63 | 26.7121962 | 20.8994752 | 47.6116714 | 0.50405442 | 0.39436941 | 0.89842383 | 0.7823945 | | *p*=0.41683 | |
| JCM 9941 | *A. gamsii* | Sab | 15 | 105 | 16.49 | 6.038 | 70.11 | 16.49 | 6.038 | 22.528 | 0.73197798 | 0.26802202 | 1 | 0.36616131 | JCM 9941 | *p*=0.11123 | Not affected |
| | | Sab+Mg | 15 | 73 | 9.28066667 | 12.5106667 | 62.06 | 10.4844915 | 14.1334651 | 24.6179565 | 0.46539824 | 0.62737327 | 1.09277151 | 1.34803535 | | *p*=0.5 | |
| JCM 10018 | *A. gracile* | Sab | 15 | 86 | 11.1466667 | 34.3466667 | 87.43 | 11.1466667 | 34.3466667 | 45.4933334 | 0.24501759 | 0.75498241 | 1 | 3.08133971 | JCM 10018 | ***p*=0.00755** | decreased |
| | | Sab+Mg | 15 | 84 | 11.7706667 | 7.684 | 58.13 | 17.7035849 | 11.5570638 | 29.2606487 | 0.3891468 | 0.25403862 | 0.64318542 | 0.65280924 | | *p*=0.08534 | |
| JCM 10691 | *A. veenhuisii* | Sab | 15 | 80 | 12.1133333 | 60.9006667 | 222.86 | 12.1133333 | 60.9006667 | 73.014 | 0.16590426 | 0.83409574 | 1 | 5.02757294 | JCM 10691 | *p*=0.31732 | Not affected |
| | | Sab+Mg | 15 | 133 | 22.5486667 | 127.118 | 353.92 | 14.1986773 | 80.0449748 | 94.2436521 | 0.19446513 | 1.09629626 | 1.29076139 | 5.63749519 | | *p*=0.24604 | |
| HIS 16 | *C. spelunceum* | Sab | 19 | 93 | 10.5152632 | 3.02789474 | 70.33 | 10.5152632 | 3.02789474 | 13.5431579 | 0.77642624 | 0.22357376 | 1 | 0.28795235 | HIS 16 | *p*=0.08226 | decreased |
| | | Sab+Mg | 20 | 107 | 4.281 | 0 | 48.47 | 6.21173365 | 0 | 6.21173365 | 0.45866213 | 0 | 0.45866213 | 0 | | ***p*<0.00001** | |
| HIS 19 | *C. cavernicola* | Sab | 15 | 123 | 14.1373333 | 12.4653333 | 93.68 | 14.1373333 | 12.4653333 | 26.6026666 | 0.53142542 | 0.46857458 | 1 | 0.88173159 | HIS 19 | *p*=0.14917 | Not affected |
| | | Sab+Mg | 15 | 204 | 26.5666667 | 16.718 | 154.64 | 16.09393 | 10.1276658 | 26.2215958 | 0.60497432 | 0.38070115 | 0.98567547 | 0.62928482 | | *p*=0.43251 | |
| JCM 1462 | *C. cutaneum* | Sab | 15 | 288 | 27.714 | 16.8173333 | 140.34 | 27.714 | 16.8173333 | 44.5313333 | 0.62234831 | 0.37765169 | 1 | 0.60681725 | JCM 1462 | *p*=0.48405 | Not affected |
| | | Sab+Mg | 15 | 359 | 39.8566667 | 27.654 | 171.32 | 32.6493381 | 22.6532942 | 55.3026323 | 0.73317675 | 0.5087046 | 1.24188135 | 0.69383625 | | *p*=0.38591 | |
| JCM 1532 | *C. curvatum* | Sab | 16 | 76 | 9.050625 | 2.409375 | 37.24 | 9.050625 | 2.409375 | 11.46 | 0.78975785 | 0.21024215 | 1 | 0.2662109 | JCM 1532 | *p*=0.18673 | Not affected |
| | | Sab+Mg | 16 | 66 | 7.8325 | 0 | 21.99 | 13.2643156 | 0 | 13.2643156 | 1.15744464 | 0 | 1.15744464 | 0 | | *p*=0.16853 | |
| JCM 9939 | *C. mucoides* | Sab | 15 | 109 | 15.6986667 | 1.63733333 | 59.29 | 15.6986667 | 1.63733333 | 17.336 | 0.90555299 | 0.09444701 | 1 | 0.1042976 | JCM 9939 | *p*=0.27425 | Not affected |
| | | Sab+Mg | 15 | 128 | 15.748 | 0 | 56.66 | 16.4789785 | 0 | 16.4789785 | 0.95056405 | 0 | 0.95056405 | 0 | | *p*=0.14007 | |

| | | | | | | | | | | | | | | | | |
|---|---|---|---|---|---|---|---|---|---|---|---|---|---|---|---|---|
| JCM 11170 | *C. dermatis* | Sab | 15 | 89 | 14.9913333 | 4.07533333 | 53.9 | 14.9913333 | 4.07533333 | 19.0666666 | 0.78625874 | 0.21374126 | 1 | 0.27184596 | JCM 11170 | ***p*=0.00289** | Increased |
| | | Sab+Mg | 15 | 92 | 13.652 | 19.5406667 | 80.93 | 9.09233659 | 13.0142337 | 22.1065703 | 0.4768708 | 0.68256471 | 1.15943551 | 1.43134095 | | *p*=0.40129 | |
| JCM 31833 | *C. cyanovorans* | Sab | 13 | 41 | 5.04615385 | 0 | 23.27 | 5.04615385 | 0 | 5.04615385 | 1 | 0 | 1 | 0 | JCM 31833 | *p*=0.49202 | Not affected |
| | | Sab+Mg | 15 | 45 | 4.69333333 | 0 | 18.03 | 6.05734146 | 0 | 6.05734146 | 1.20038779 | 0 | 1.20038779 | 0 | | *p*=0.10749 | |
| JCM 11166 | *C. daszewskae* | Sab | 16 | 72 | 11.3 | 1.50875 | 41.88 | 11.3 | 1.50875 | 12.80875 | 0.88220943 | 0.11779057 | 1 | 0.1335177 | JCM 11166 | *p*=0.40517 | Not affected |
| | | Sab+Mg | 16 | 52 | 6.25875 | 1.03875 | 29.21 | 8.97351763 | 1.48931359 | 10.4628312 | 0.70057715 | 0.11627314 | 0.8168503 | 0.16596765 | | *p*=0.0505 | |
| JCM 14201 | *C. arboriforme* | Sab | 21 | 60 | 4.56571429 | 0 | 18.69 | 4.56571429 | 0 | 4.56571429 | 1 | 0 | 1 | 0 | JCM 14201 | *p*=0.49601 | Not affected |
| | | Sab+Mg | 22 | 84 | 4.12181818 | 0 | 19.14 | 4.02491023 | 0 | 4.02491023 | 0.88155105 | 0 | 0.88155105 | 0 | | *p*=0.34458 | |
| JCM 12878 | *Ta. koratensis* | Sab | 20 | 66 | 4.9975 | 0 | 20.56 | 4.9975 | 0 | 4.9975 | 1 | 0 | 1 | 0 | JCM 12878 | *p*=0.49601 | Not affected |
| | | Sab+Mg | 20 | 77 | 4.2065 | 0 | 18.95 | 4.56388602 | 0 | 4.56388602 | 0.91323382 | 0 | 0.91323382 | 0 | | *p*=0.31918 | |
| JCM 11965 | *Ta. tepidaria* | Sab | 16 | 42 | 6.72375 | 0 | 21.61 | 6.72375 | 0 | 6.72375 | 1 | 0 | 1 | 0 | JCM 11965 | *p*=0.49202 | decreased |
| | | Sab+Mg | 17 | 64 | 2.67411765 | 0 | 22.97 | 2.5157894 | 0 | 2.5157894 | 0.37416462 | 0 | 0.37416462 | 0 | | ***p*<0.00001** | |
| JCM 10690 | *P. guehoae* | Sab | 12 | 63 | 14.37 | 53.3266667 | 253.11 | 14.37 | 53.3266667 | 67.6966667 | 0.21227042 | 0.78772958 | 1 | 3.71097193 | JCM 10690 | ***p*=0.04093** | Increased |
| | | Sab+Mg | 16 | 110 | 17.6725 | 114.6325 | 247.09 | 18.1030656 | 117.425359 | 135.528425 | 0.26741443 | 1.73458111 | 2.00199554 | 6.48649031 | | *p*=0.06552 | |
| JCM 1530 | *Pr. fragicola* | Sab | 16 | 47 | 6.969375 | 0.72125 | 29.55 | 6.969375 | 0.72125 | 7.690625 | 0.90621698 | 0.09378302 | 1 | 0.10348848 | JCM 1530 | *p*=0.5 | Not affected |
| | | Sab+Mg | 16 | 50 | 5.743125 | 0.74 | 20.72 | 8.19060539 | 1.05535714 | 9.24596254 | 1.06501167 | 0.13722645 | 1.20223812 | 0.12884971 | | *p*=0.06811 | |
| JCM 1457 | *V. humicola* | Sab | 16 | 81 | 7.66 | 17.054375 | 54.58 | 7.66 | 17.054375 | 24.714375 | 0.30994108 | 0.69005892 | 1 | 2.22641971 | JCM 1457 | *p*=0.11123 | Not affected |
| | | Sab+Mg | 16 | 83 | 8.91125 | 10.01875 | 49.5 | 9.82577828 | 11.0469369 | 20.8727152 | 0.39757341 | 0.44698427 | 0.84455768 | 1.12428111 | | *p*=0.1335 | |

Fig. 8A

| Strains | | Media | Cell number | Average length (*μm*) | Standard deviation (*μm*) | Relative value | Statics *p*-value | Impact by magnesium addition |
|---|---|---|---|---|---|---|---|---|
| JCM 1943 | *D. reessii* | Sab | 328 | 24.91 | 25.87 | 1 | ***p*< 0.00001** | Increased |
| | | Sab+Mg | 358 | 72.11 | 53.34 | 2.89482136 | | |
| JCM 6359 | *D. geotrichum* | Sab | 369 | 55.95 | 83.94 | 1 | ***p*=0.04236** | Increased |
| | | Sab+Mg | 293 | 106.02 | 189.34 | 1.89490617 | | |
| JCM 9886 | *T. pullulans* | Sab | 394 | 19.17 | 20 | 1 | ***p*< 0.00001** | Increased |
| | | Sab+Mg | 391 | 91.49 | 54.33 | 4.77256129 | | |
| BY1438 | *S. cerevisiae* | Sab | 185 | 5.52 | 1.52 | 1 | ***p*< 0.00001** | decreased |
| | | Sab+Mg | 233 | 4.56 | 1.34 | 0.82608696 | | |
| FY7507 | *S. pombe* | Sab | 356 | 11.64 | 2.7 | 1 | ***p*< 0.00001** | decreased |
| | | Sab+Mg | 231 | 10.19 | 2.51 | 0.87542955 | | |

Fig. 8B

| Strains | | Media | Cell number | Average cellular area ($\mu m^2$) | Average number of LDs | LD number per unit area | Ratio | Statics *p*-value | Impact by magnesium addition |
|---|---|---|---|---|---|---|---|---|---|
| JCM 1943 | *D. reessii* | Sab | 14 | 140.29 | 5 | 0.03564167 | 1 | ***p*=0.0088** | Increased |
| | | Sab+Mg | 15 | 274.45 | 23.4 | 0.08526125 | 2.39217887 | | |
| JCM 6359 | *D. geotrichum* | Sab | 15 | 442.77 | 12.6 | 0.02845697 | 1 | ***p*=0.00084** | Increased |
| | | Sab+Mg | 16 | 377.19 | 27.125 | 0.07191429 | 2.52712393 | | |
| JCM 9886 | *T. pullulans* | Sab | 15 | 261.56 | 20 | 0.07646549 | 1 | ***p*=0.00008** | Increased |
| | | Sab+Mg | 15 | 391.19 | 56.1333333 | 0.14349216 | 1.87656105 | | |
| BY1438 | *S. cerevisiae* | Sab | 17 | 19.82 | 7.82352941 | 0.39468657 | 1 | ***p*< 0.00001** | Increased |
| | | Sab+Mg | 17 | 10.95 | 7.94117647 | 0.72530164 | 1.83766486 | | |
| FY7507 | *S. pombe* | Sab | 17 | 50.33 | 15.5882353 | 0.3096959 | 1 | *p*=0.21498 | Not affected |
| | | Sab+Mg | 17 | 32.78 | 10.8823529 | 0.3320051 | 1.07203583 | | |

Fig. 8C

| Strains | | Media | Cell number | No septum | Single septum | Multi-septa | Rate of septum cells (%) | Increase rate of septation (%) | Rate of multi-septa cells (%) | Increase rate of multi-septation (%) | Impact by magnesium addition |
|---|---|---|---|---|---|---|---|---|---|---|---|
| JCM 1943 | *D. reessii* | Sab | 316 | 311 | 5 | 0 | 1.58227848 | 20.3402299 | 0 | 28.57 | Increased |
| | | Sab+Mg | 87 | 59 | 20 | 8 | 32.183908 | | 28.5714286 | | |
| JCM 6359 | *D. geotrichum* | Sab | 170 | 131 | 30 | 9 | 22.9411765 | 1.32215336 | 23.0769231 | 1.625 | Increased |
| | | Sab+Mg | 211 | 147 | 40 | 24 | 30.3317536 | | 37.5 | | |
| JCM 9886 | *T. pullulans* | Sab | 120 | 66 | 36 | 18 | 45 | 1.5 | 33.3333333 | 2.18518519 | Increased |
| | | Sab+Mg | 120 | 39 | 22 | 59 | 67.5 | | 72.8395062 | | |
| BY1438 | *S. cerevisiae* | Sab | 219 | 195 | 24 | 0 | 10.9589041 | 0.04073661 | 0 | 0 | Not affected |
| | | Sab+Mg | 224 | 223 | 1 | 0 | 0.44642857 | | 0 | | |
| FY7507 | *S. pombe* | Sab | 160 | 139 | 21 | 0 | 13.125 | 0.61943477 | 0 | 0 | Not affected |
| | | Sab+Mg | 246 | 226 | 20 | 0 | 8.1300813 | | 0 | | |

Fig. 8D

| Strains | | Media | Cell number | Vacuolar number | Average vacuole area less than 10 $\mu m^2$ | Average vacuole area more than 10 $\mu m^2$ | Average cellular area ($\mu m^2$) | Corrected by cellular area | | Total corrected area of vacuoles ($\mu m^2$) | Ratio | | Total ratios of vacuole area described in Fig. 8D | Relative values of larger vacuoles | Strains | Statics p-values Upper: relative value of vacuoles ≥10 $\mu m^2$ (*) Lower: total corrected area of vacuoles(**) | Impact by magnesium addition |
|---|---|---|---|---|---|---|---|---|---|---|---|---|---|---|---|---|---|
| | | | | | | | | Average vacuole area less than 10 $\mu m^2$ | Average vacuole area more than 10 $\mu m^2$ | | Vacuole area less than 10 $\mu m^2$ | Vacuole area more than 10 $\mu m^2$ | | | | | |
| JCM 1943 | *D. reessii* | Sab | 14 | 80 | 21.5192857 | 18.405 | 140.29 | 21.5192857 | 18.405 | 39.9242857 | 0.5390024 | 0.4609976 | 1 | 0.85527932 | JCM 1943 | ***p* =0.00226** | Increased |
| | | Sab+Mg | 15 | 132 | 23.726 | 64.1013333 | 274.45 | 12.127967 | 32.7665369 | 44.8945039 | 0.30377418 | 0.82071692 | 1.1244911 | 2.70173368 | | *p* =0.38974 | |
| JCM 6359 | *D. geotrichum* | Sab | 15 | 96 | 7.28266667 | 176.602 | 442.77 | 7.28266667 | 176.602 | 183.884667 | 0.03960453 | 0.96039547 | 1 | 24.2496338 | JCM 6359 | ***p* =0.00539** | decreased |
| | | Sab+Mg | 16 | 155 | 23.211875 | 148.696875 | 377.19 | 27.2475991 | 174.550002 | 201.797601 | 0.14817766 | 0.94923631 | 1.09741397 | 6.40606909 | | *p* =0.33724 | |
| JCM 9886 | *T. pullulans* | Sab | 15 | 132 | 23.8306667 | 64.922 | 261.56 | 23.8306667 | 64.922 | 88.7526667 | 0.26850649 | 0.73149351 | 1 | 2.72430481 | JCM 9886 | ***p* =0.02559** | decreased |
| | | Sab+Mg | 15 | 233 | 47.5426667 | 88.22 | 391.19 | 31.7882868 | 58.9862297 | 90.7745165 | 0.35816712 | 0.6646136 | 1.02278072 | 1.85559638 | | *p* =0.48405 | |
| BY1438 | *S. cerevisiae* | Sab | 17 | 57 | 5.24588235 | 0 | 18.06 | 5.24588235 | 0 | 5.24588235 | 1 | 0 | 1 | 0 | BY1438 | *p* =0.49202 | Not affected |
| | | Sab+Mg | 17 | 44 | 4.97823529 | 0 | 16.67 | 5.39333709 | 0 | 5.39333709 | 1.02810866 | 0 | 1.02810866 | 0 | | *p* =0.40517 | |
| FY7507 | *S. pombe* | Sab | 17 | 21 | 0.40470588 | 0 | 48.97 | 0.40470588 | 0 | 0.40470588 | 1 | 0 | 1 | 0 | FY7507 | *p* =0.49202 | Increased |
| | | Sab+Mg | 17 | 126 | 7.69470588 | 0 | 29.07 | 12.9621516 | 0 | 12.9621516 | 32.0285724 | 0 | 32.0285724 | 0 | | ***p* <0.00001** | |

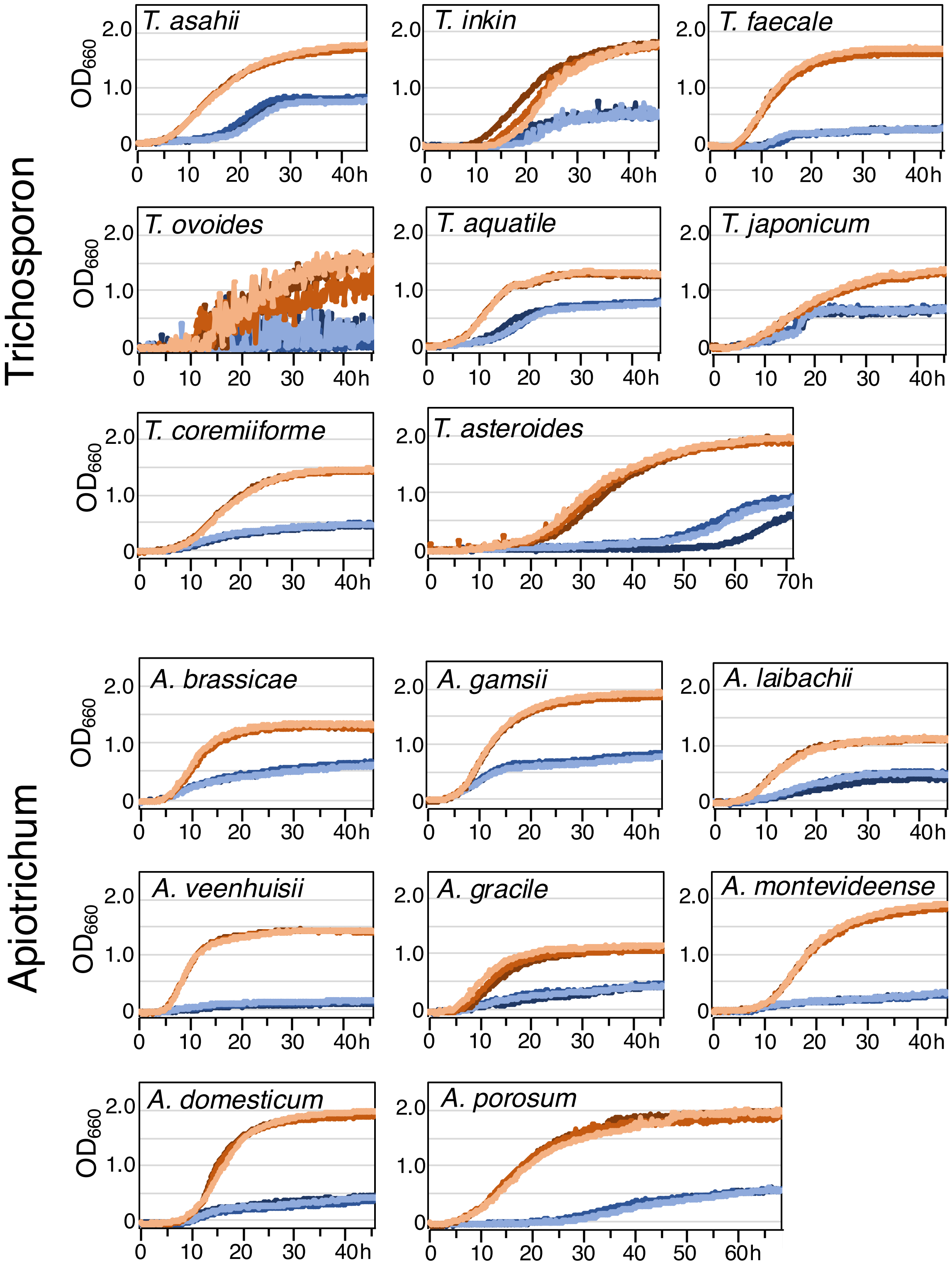


Supplemental Fig. 1

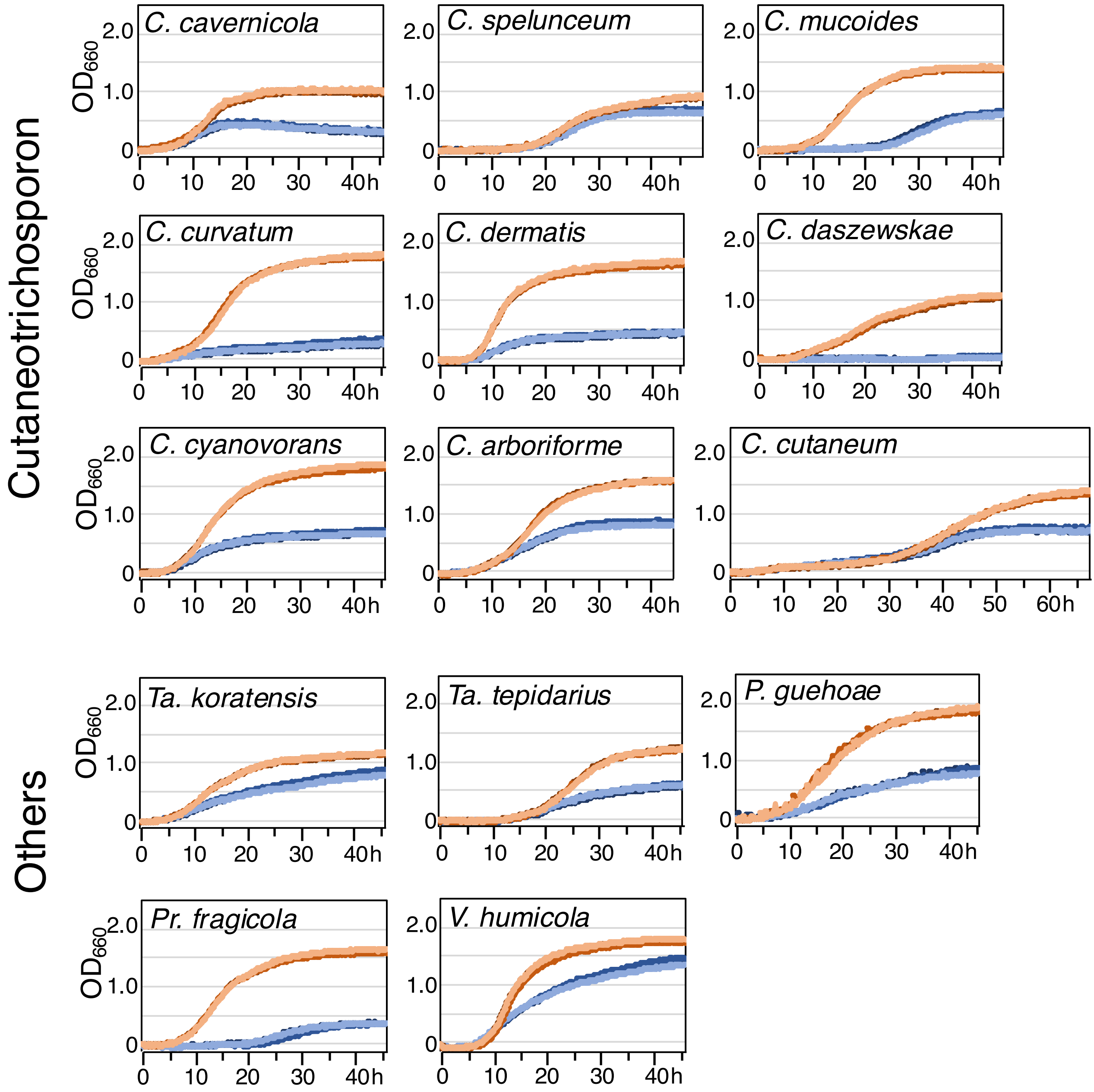


Supplemental Fig. 2

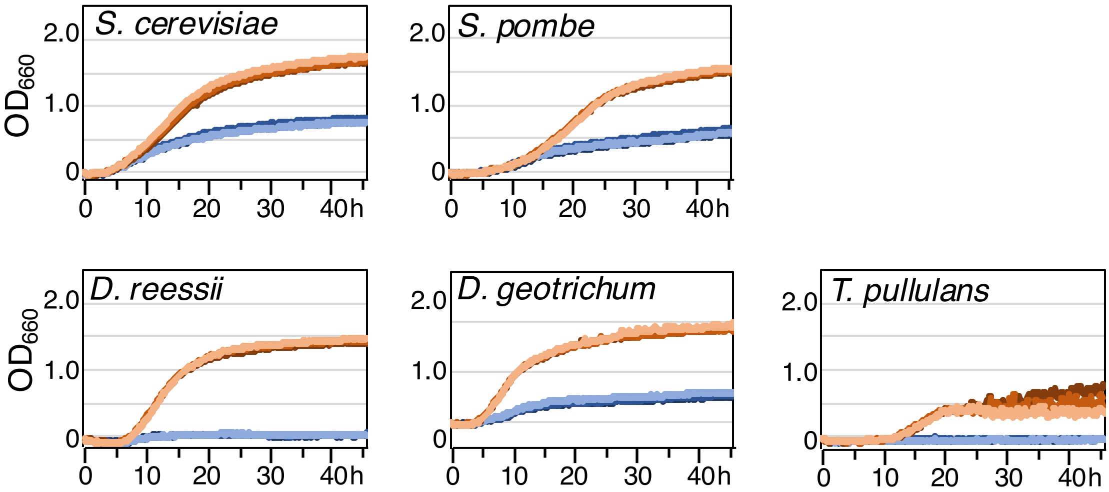


**Legends**

$OD_{660}$ values were measured in 35 yeasts using a Bio-photorecorder TVS062CA air shaker.
Blue lines indicate $OD_{660}$ values of cells cultivated in Sabouraud broth at 25˚C.
Orange lines indicate $OD_{660}$ values of cells cultivated in Sabouraud+Mg broth at 25˚C.

Supplemental fig. 1 includes results of 8 species in *Trichosporon* genus and 8 species in *Apiotrichum* genus.

Supplemental fig. 2 includes results of 9 species in *Cutaneotrichosporon* genus, 2 species in *Takashimella* genus, *P. guehoae*, *Pr. fragicola*, and *V. humicola*.

Supplemental fig. 3 includes results of *S. cerevisiae*, *Sc. pombe*, *D. reessii*, *D. geotrichum*, and *T. pullulans*.

Supplemental Fig. 3

# Supplemental Fig. 4

We observed phenotypes of lipid droplet, vacuole, and septum in 35 yeasts when magnesium was added. Cells were cultivated in Sabouraud and Sabouraud + Mg broth at 25˚C. Lipid droplet was stained with BODIPY reagent, vacuole was stained with FM4-64 reagent, septum was stained with Calcofluor White reagent. Results were shown in the order of *Trichosporon* genus (8 species), *Apiotrichum* genus (8 species), *Cutaneotrichosporon* genus (9 species), *Takashimella* genus (2 species), *P. guehoae*, *Pr. fragicola*, *V. humicola*, *D. reessii*, *D. geotrichum*, *T. pullulans*, *Sc. pombe*, *S. cerevisiae.*

# *Trichosporon* genus

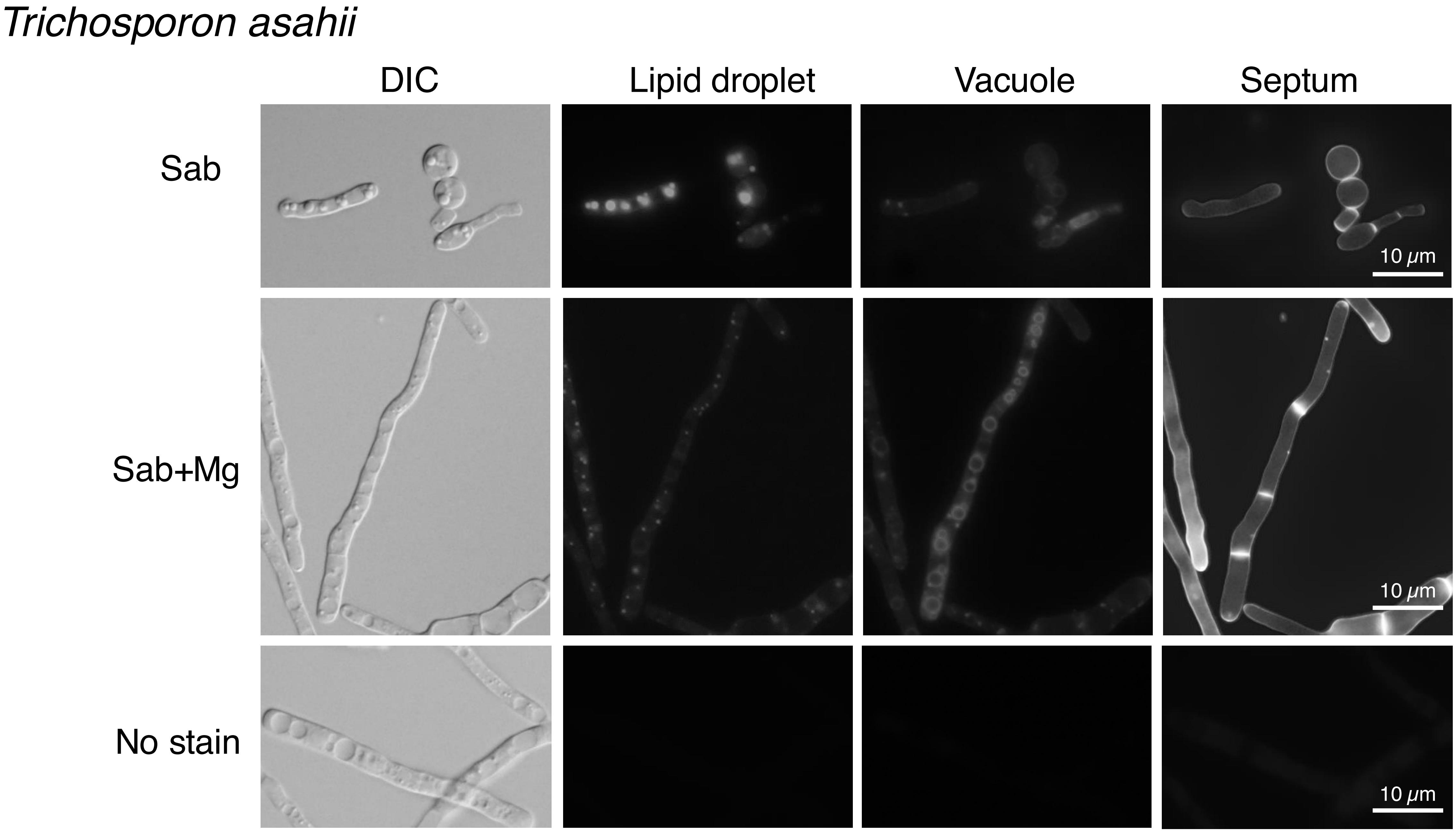
Trichosporon asahii
DIC
Lipid droplet
Vacuole
Septum
Sab
Sab+Mg
No stain
10 μm
10 μm
10 μm

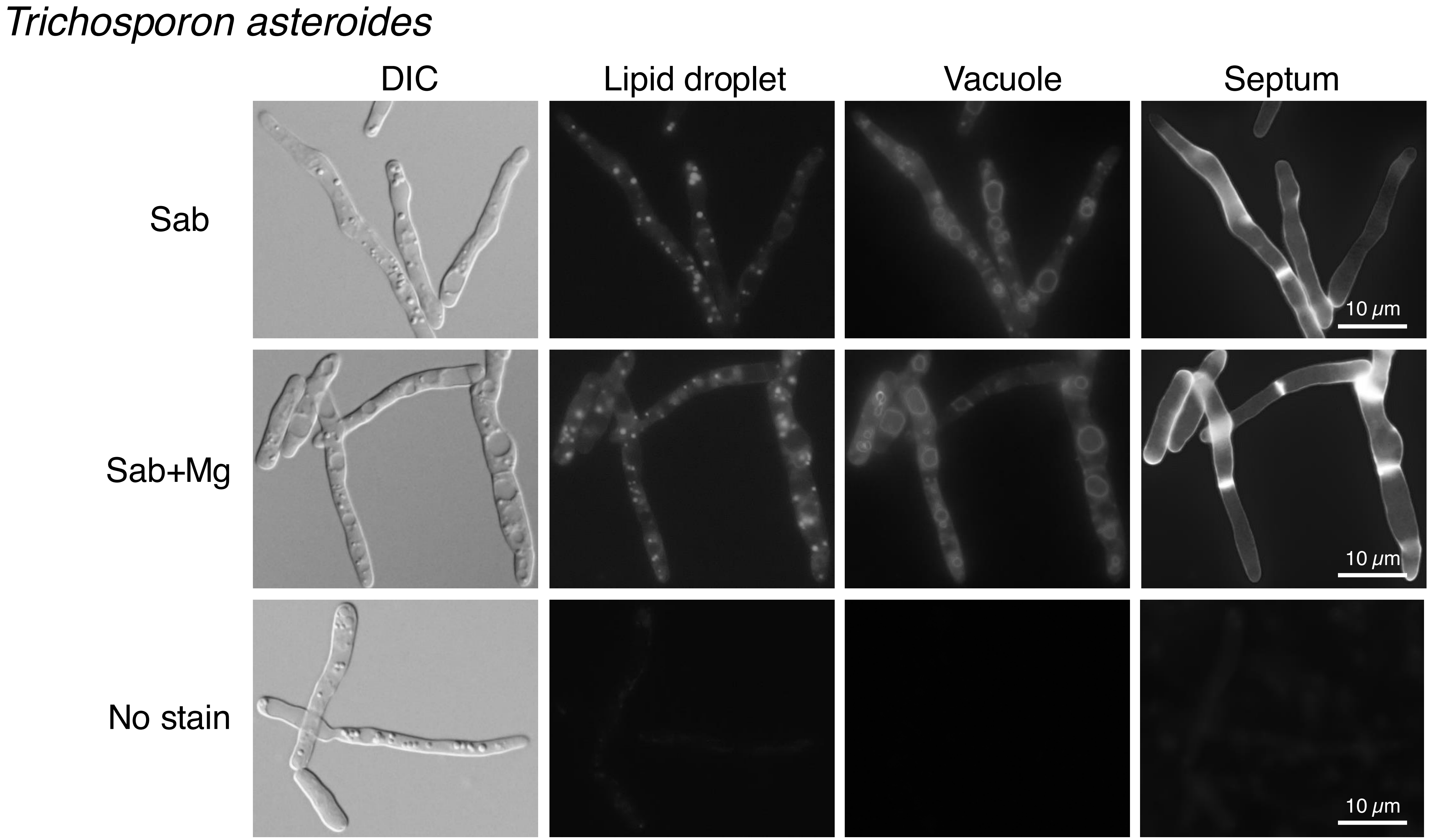
Trichosporon asteroides
DIC
Lipid droplet
Vacuole
Septum
Sab
Sab+Mg
No stain
10 μm
10 μm
10 μm

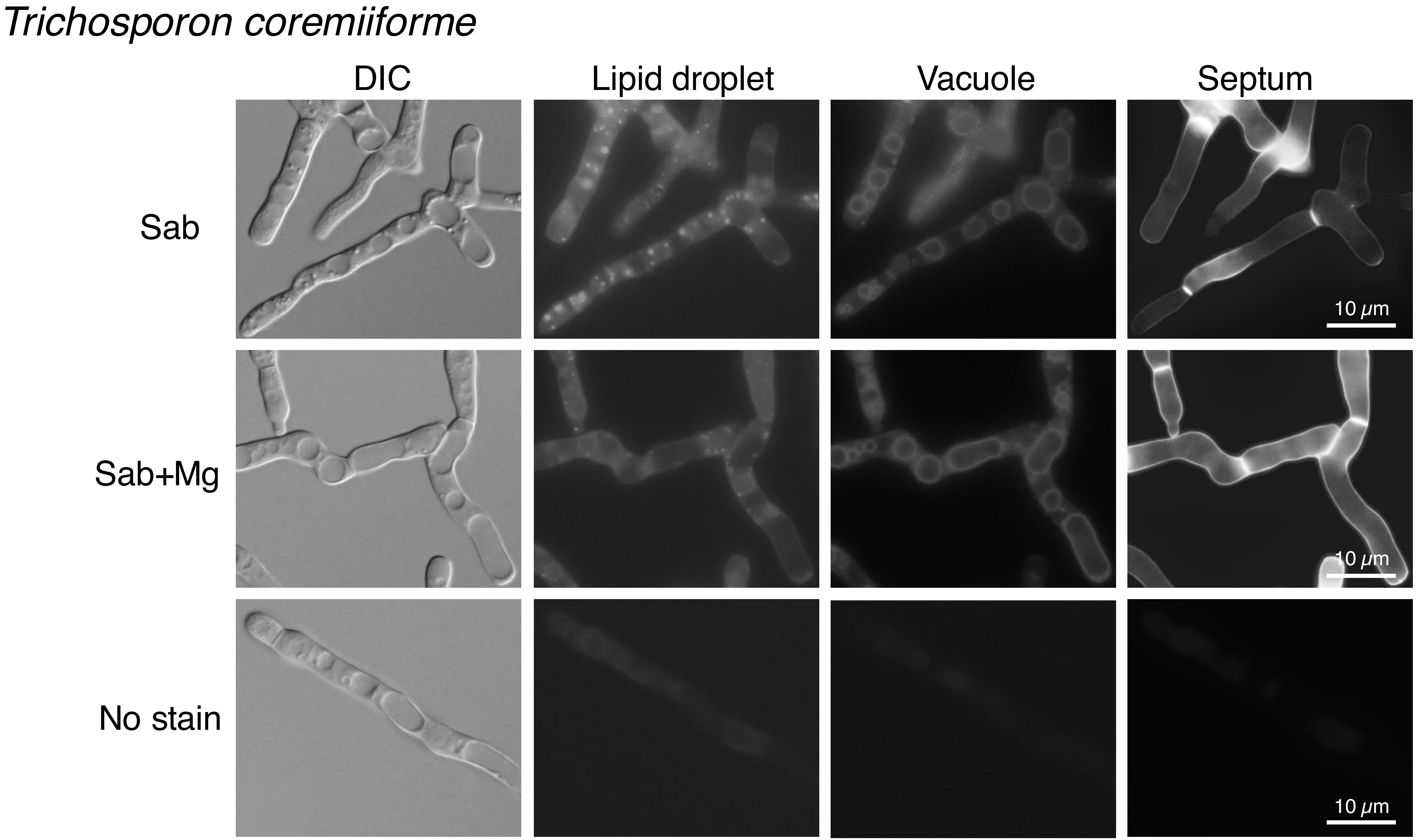

Trichosporon coremiiforme
DIC
Lipid droplet
Vacuole
Septum
Sab
Sab+Mg
No stain
10 μm
10 μm
10 μm

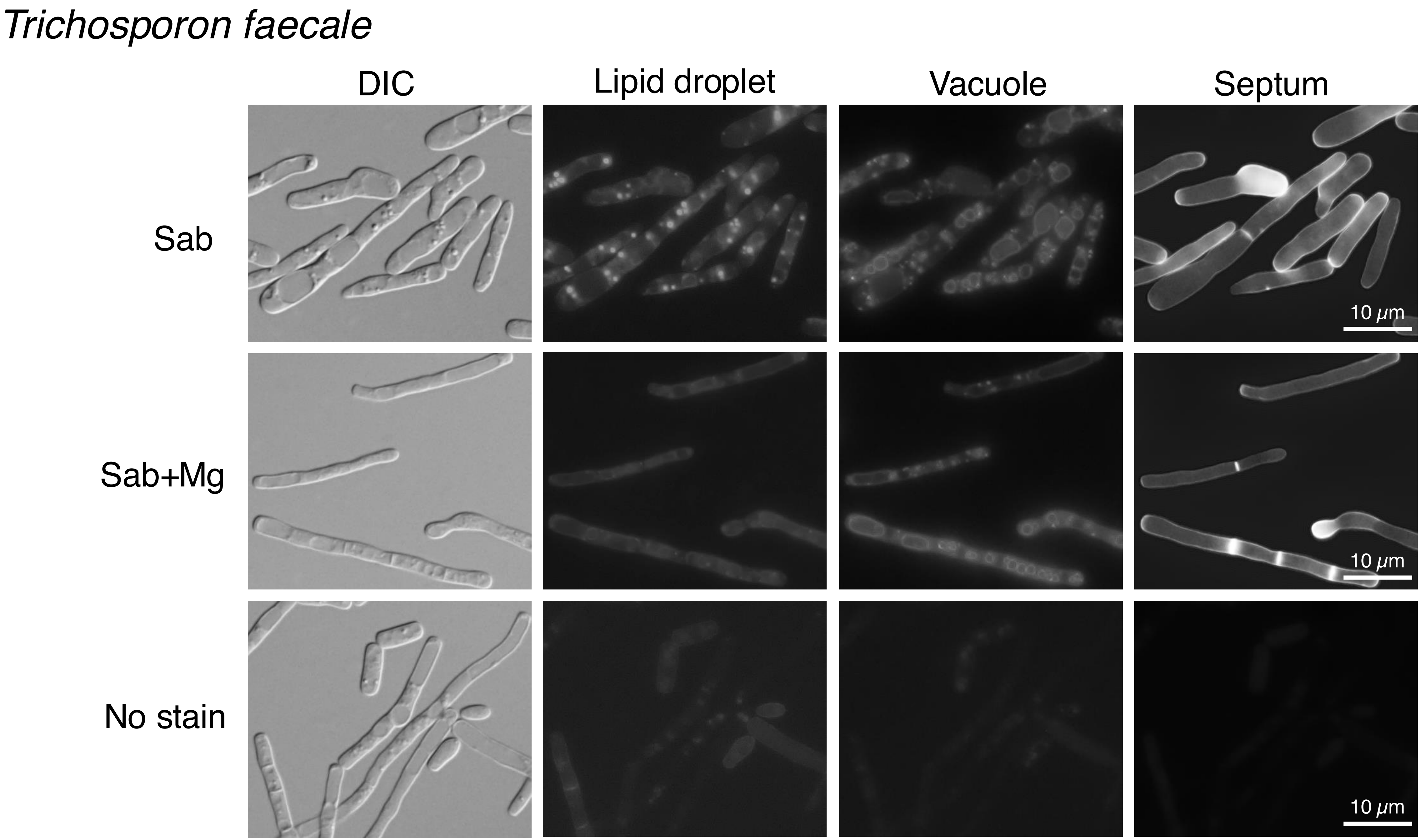

Trichosporon faecale
DIC
Lipid droplet
Vacuole
Septum
Sab
Sab+Mg
No stain
10 μm
10 μm
10 μm

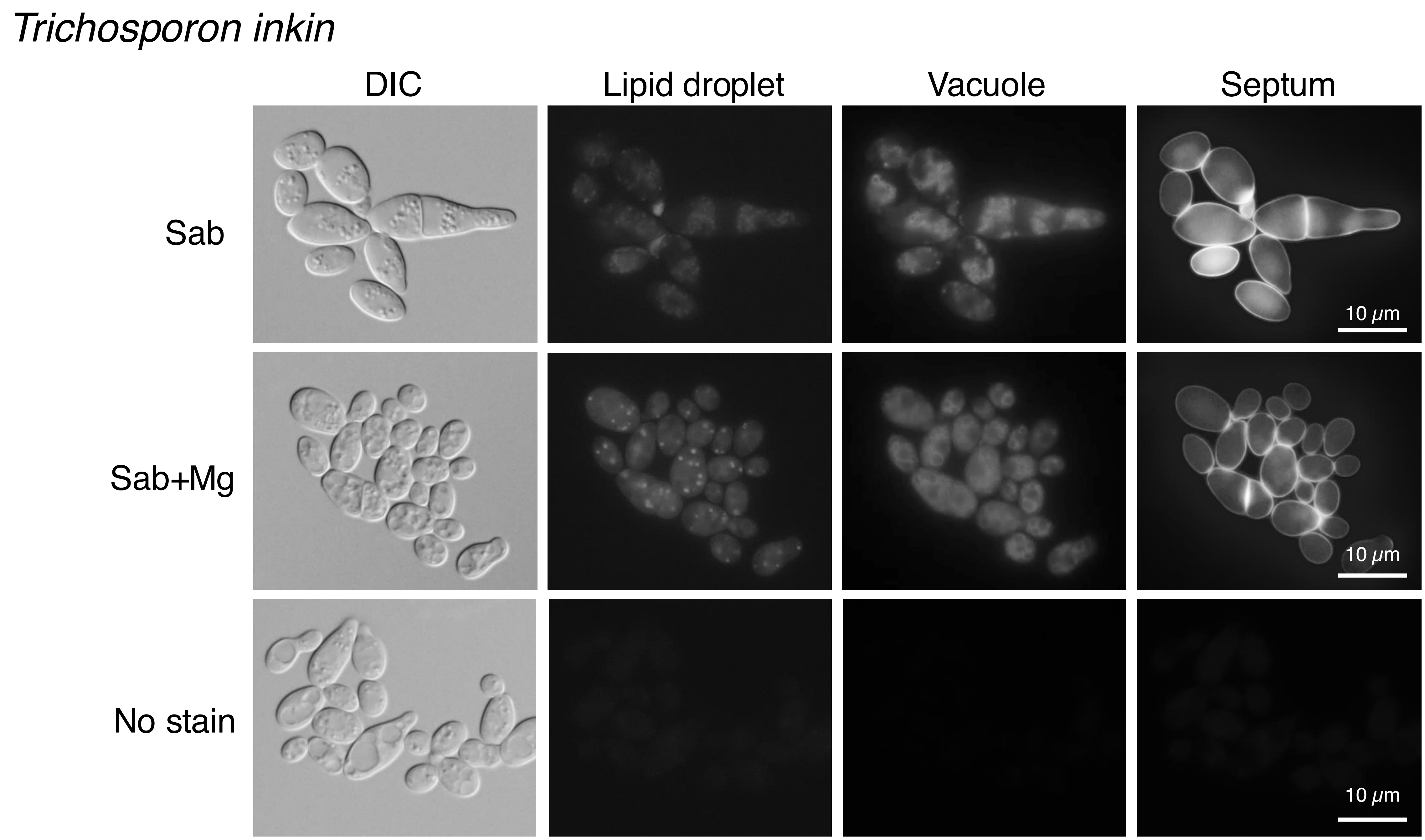
Trichosporon inkin
DIC
Lipid droplet
Vacuole
Septum
Sab
Sab+Mg
No stain
10 μm
10 μm
10 μm

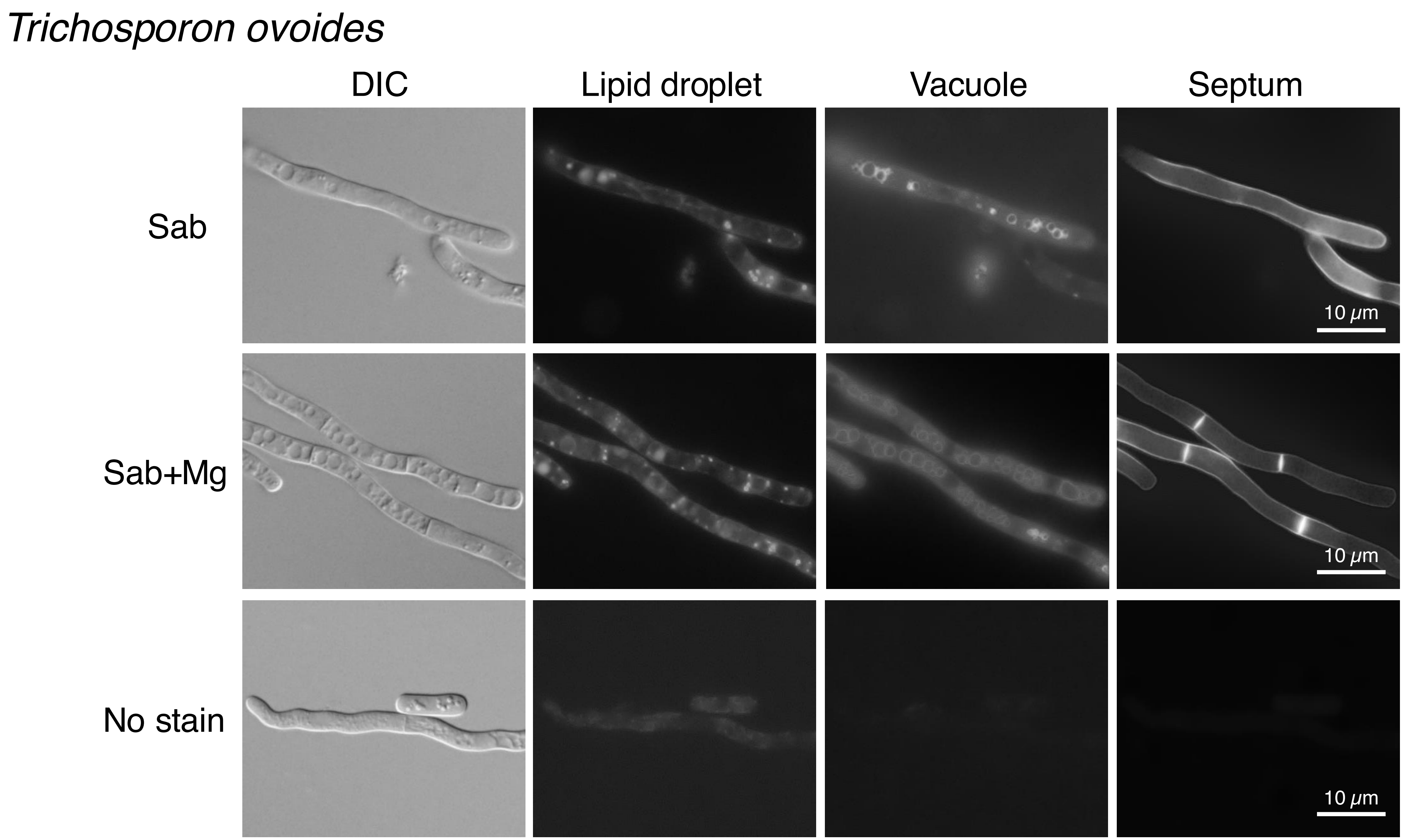
Trichosporon ovoides
DIC
Lipid droplet
Vacuole
Septum
Sab
Sab+Mg
No stain
10 μm
10 μm
10 μm

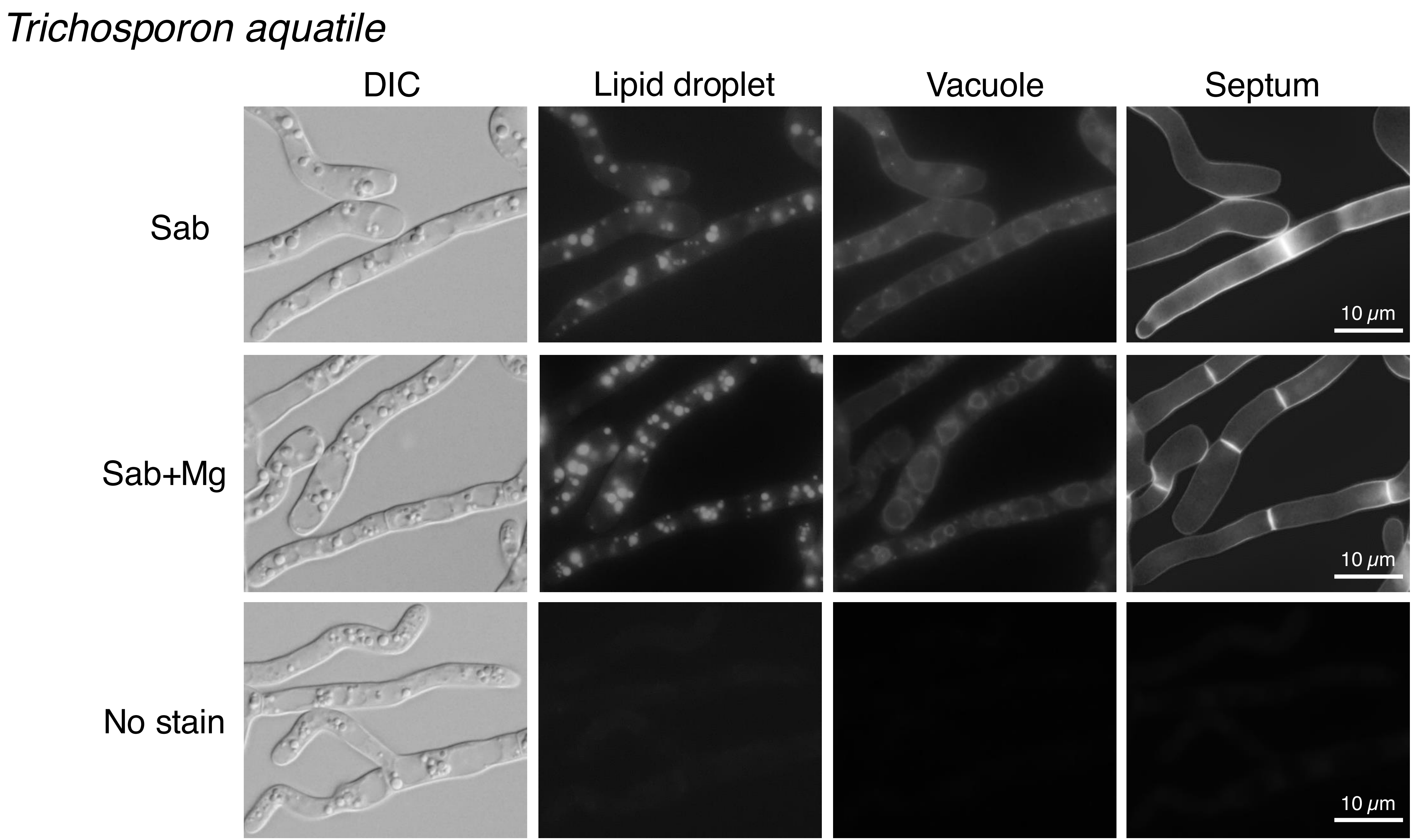

Trichosporon aquatile
DIC
Lipid droplet
Vacuole
Septum
Sab
Sab+Mg
No stain
10 μm
10 μm
10 μm

# *Trichosporon japonicum*

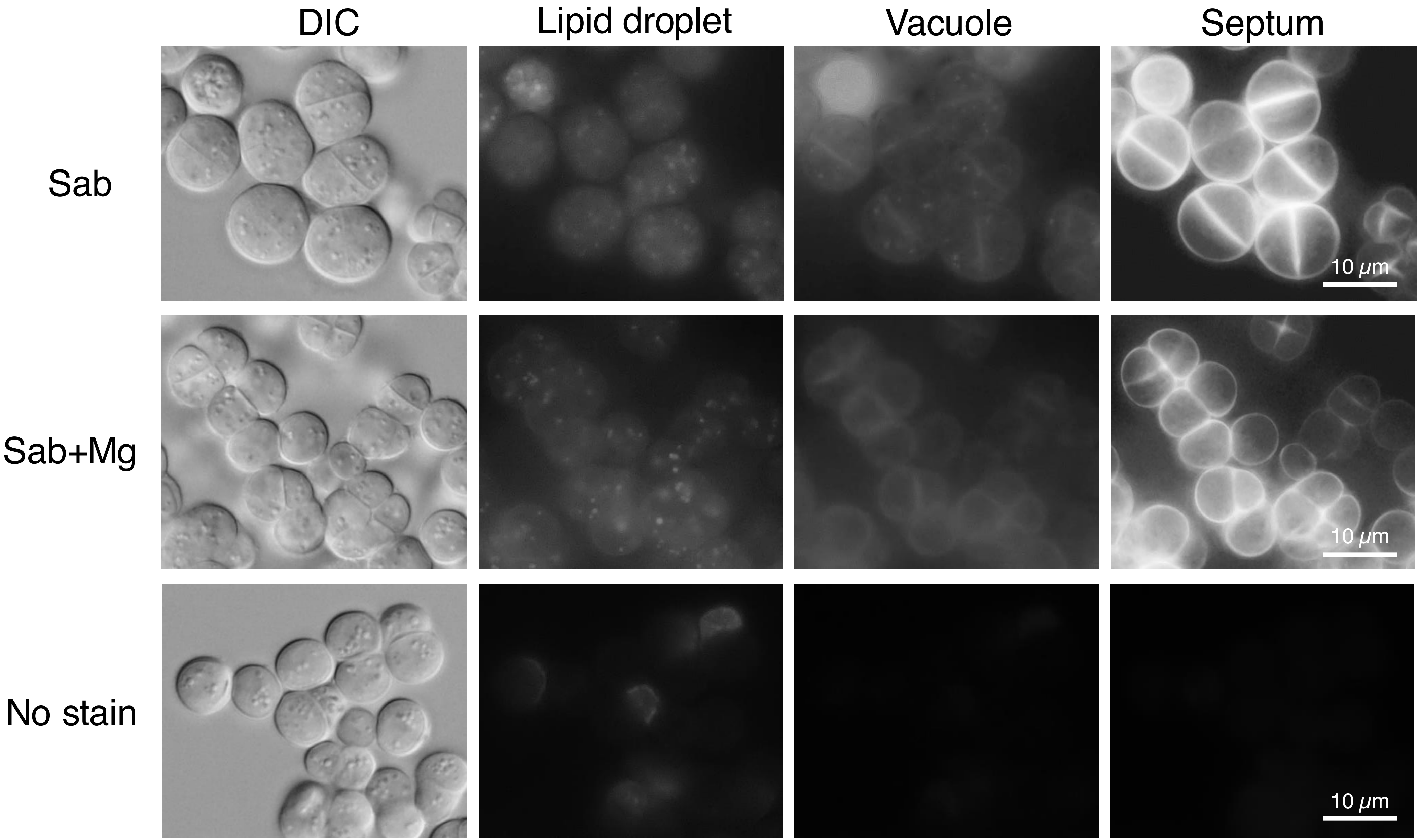

# *Apiotrichum* genus

# *Apiotrichum porosum*

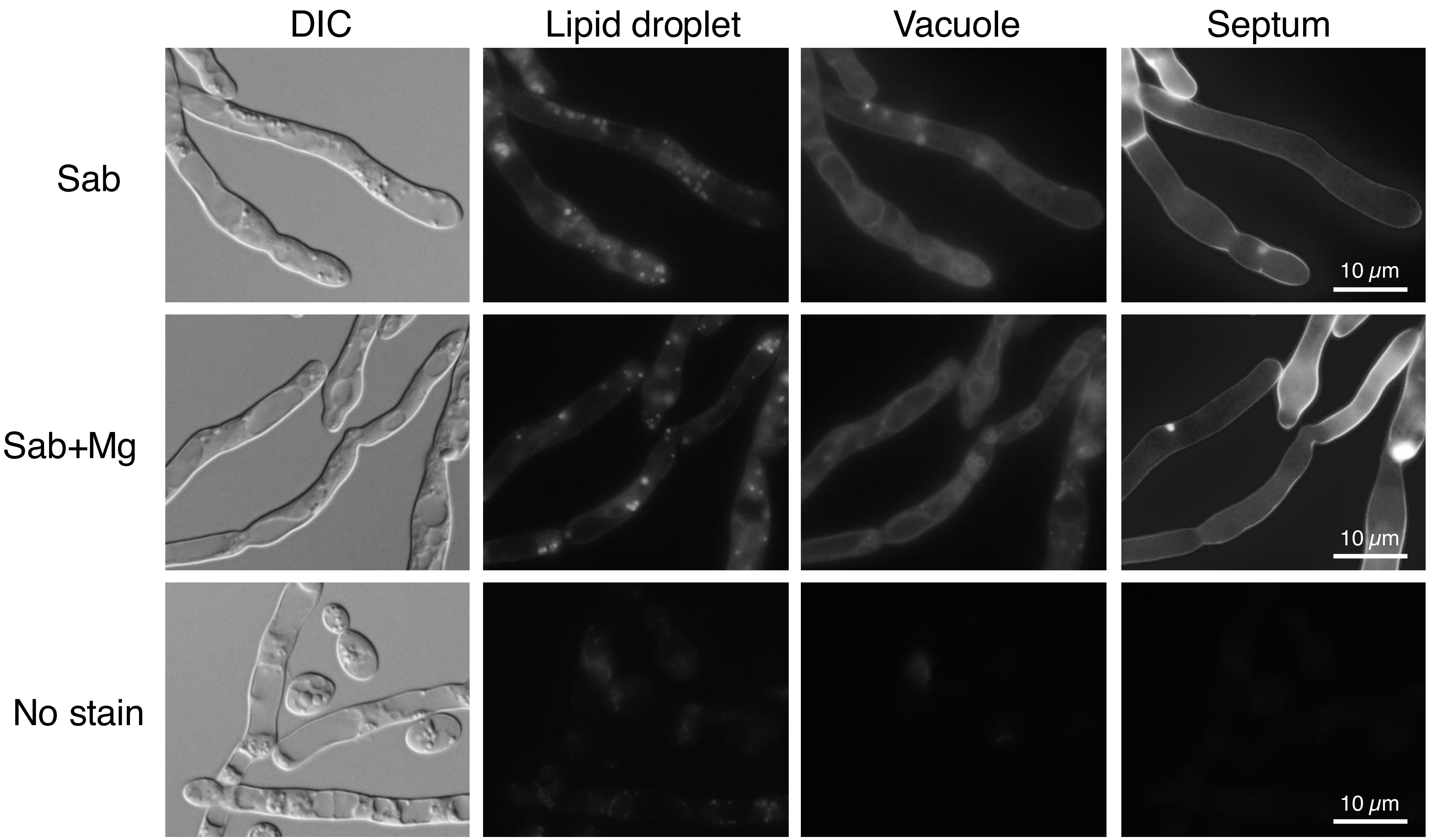

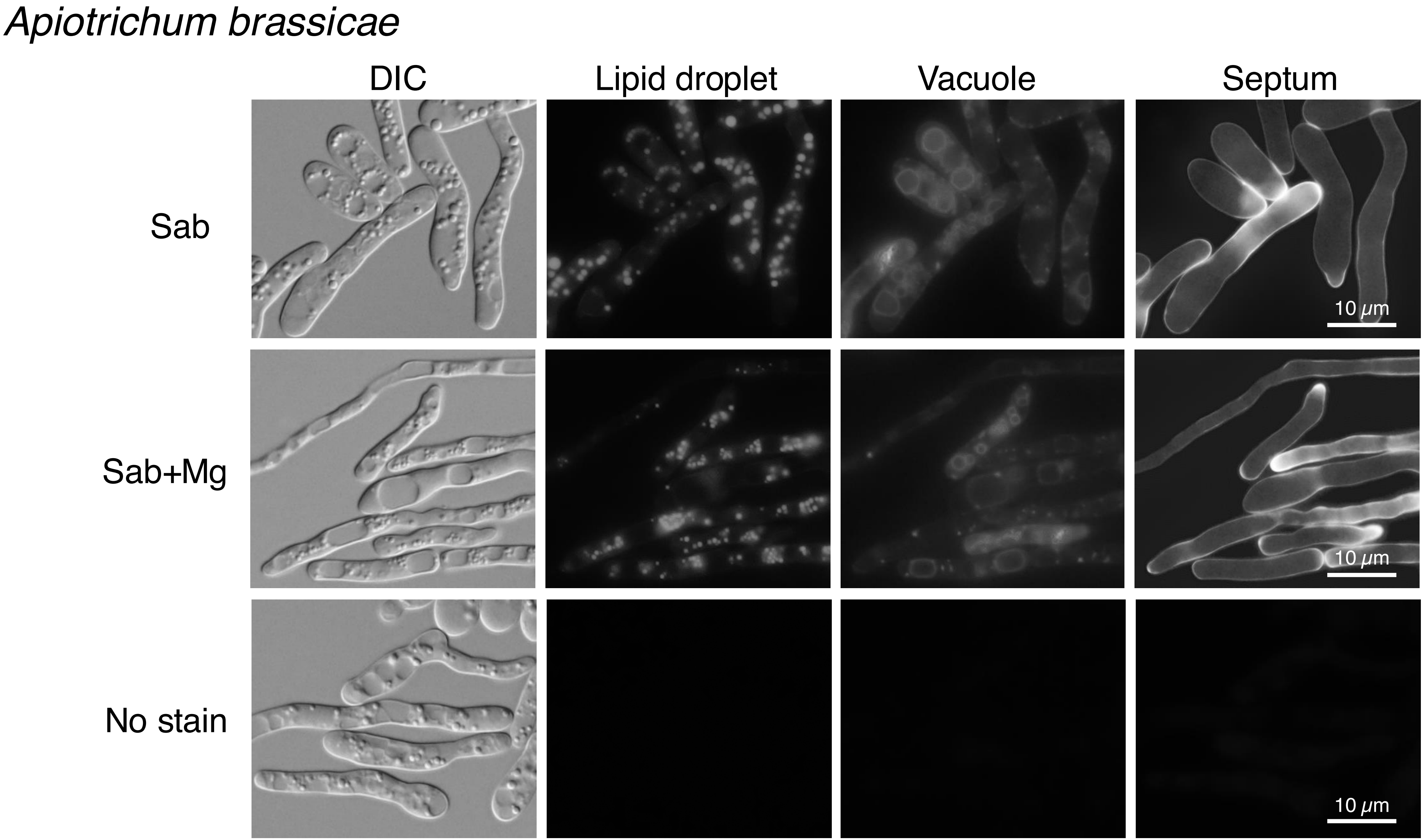
Apiotrichum brassicae
DIC
Lipid droplet
Vacuole
Septum
Sab
Sab+Mg
No stain
10 μm
10 μm
10 μm

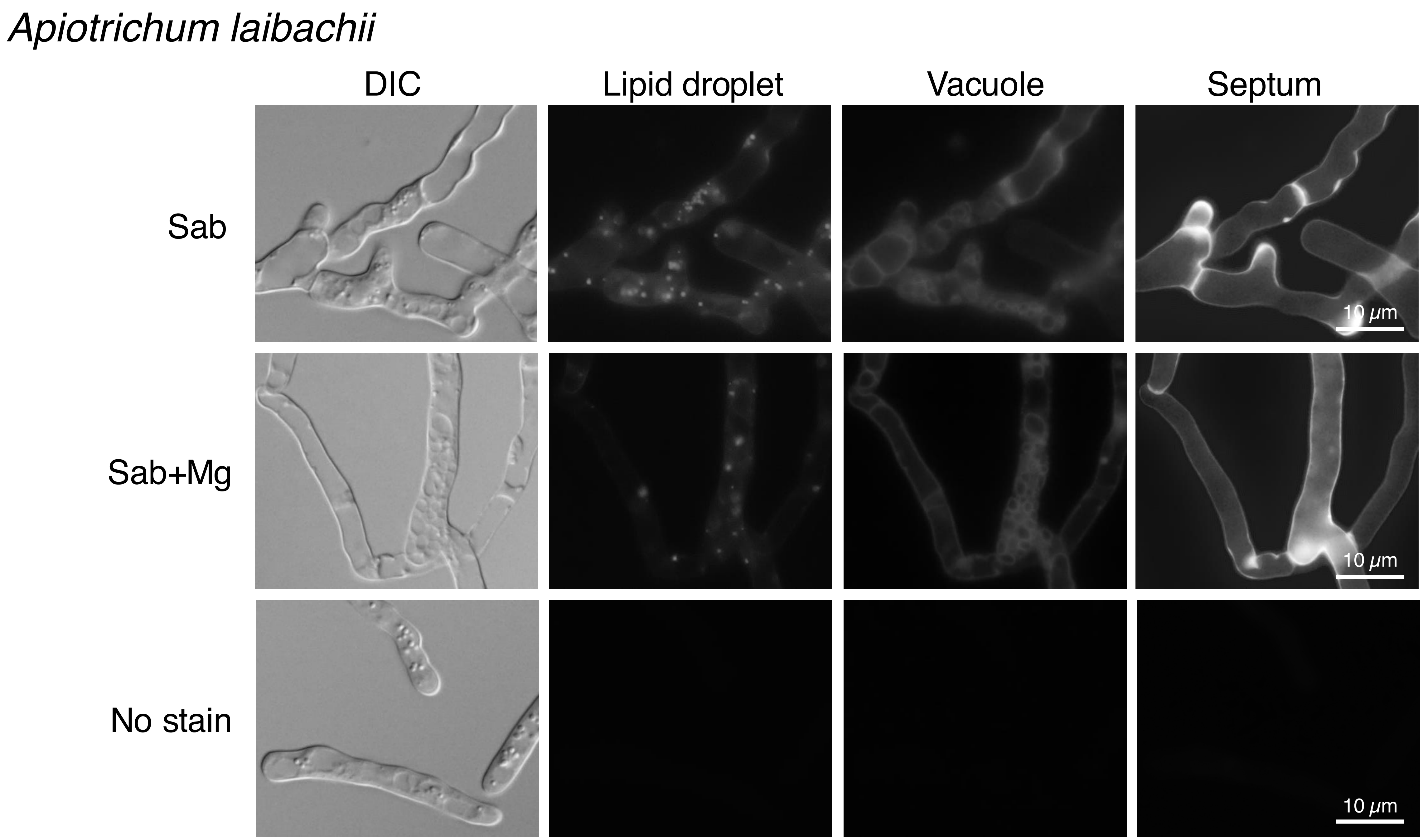
*Apiotrichum laibachii*
DIC
Lipid droplet
Vacuole
Septum
Sab
Sab+Mg
No stain
10 μm
10 μm
10 μm

*Apiotrichum domesticum*

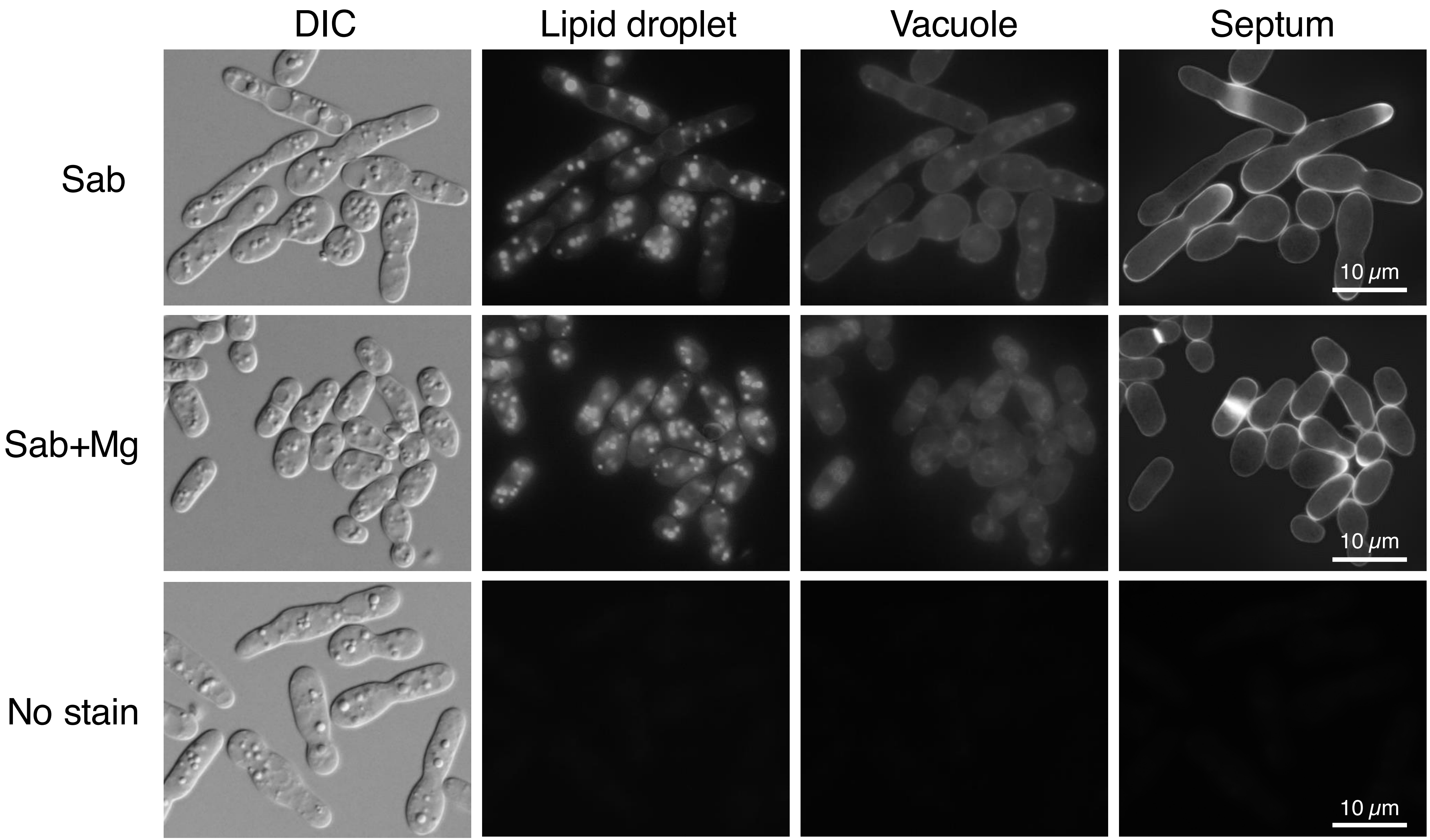

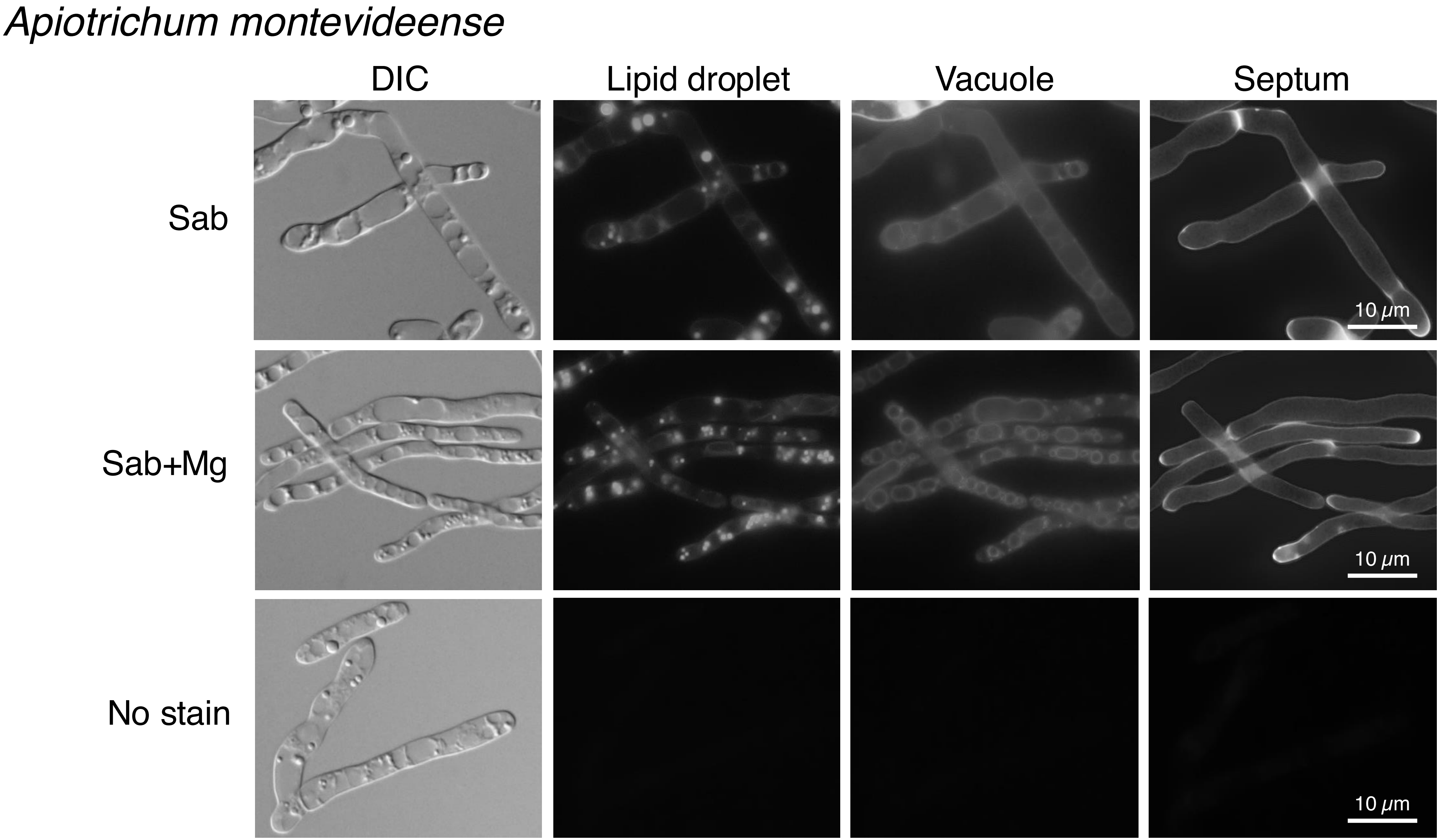

Apiotrichum montevideense
DIC
Lipid droplet
Vacuole
Septum
Sab
Sab+Mg
No stain
10 μm
10 μm
10 μm

*Apiotrichum gamsii*

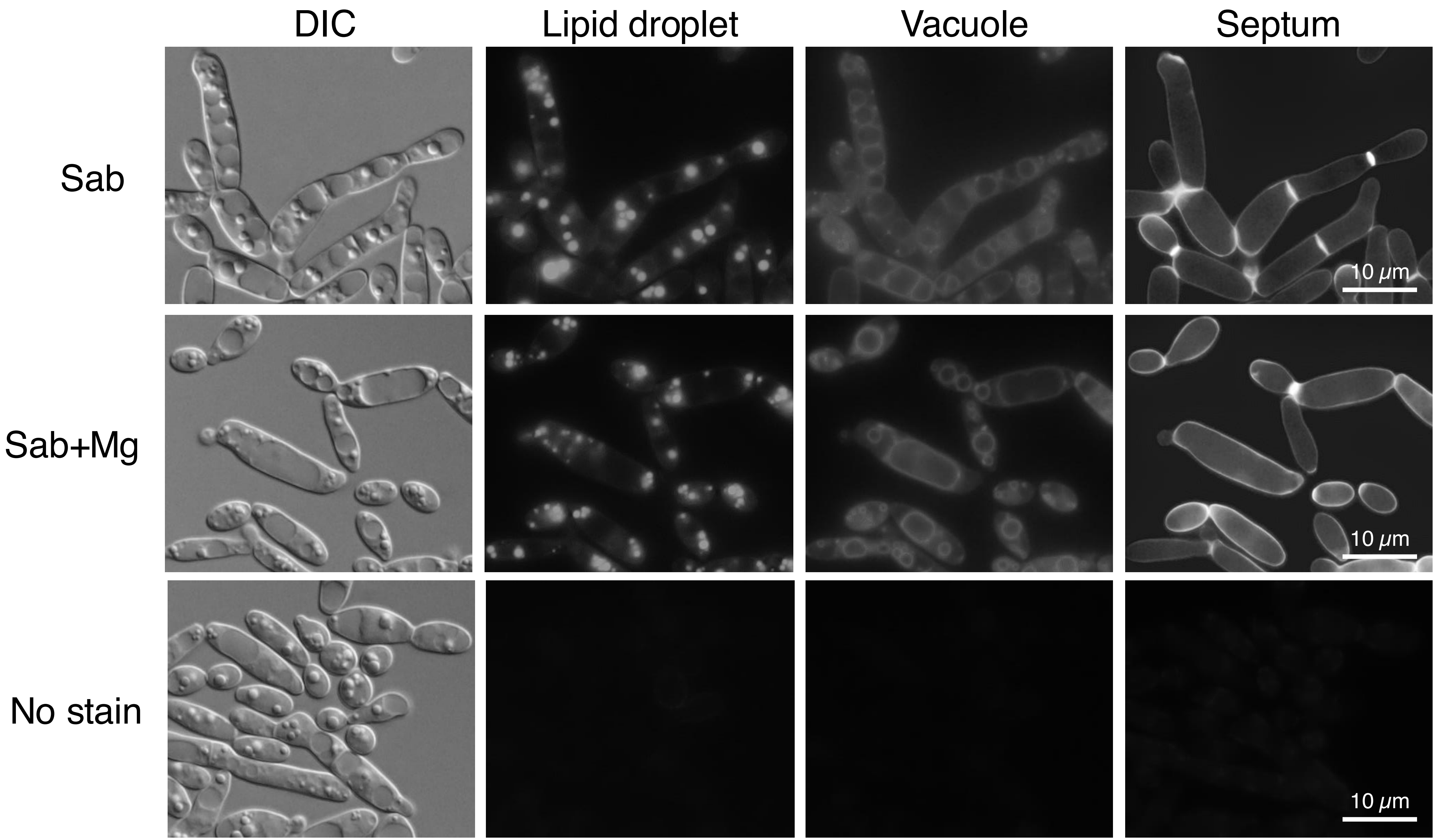

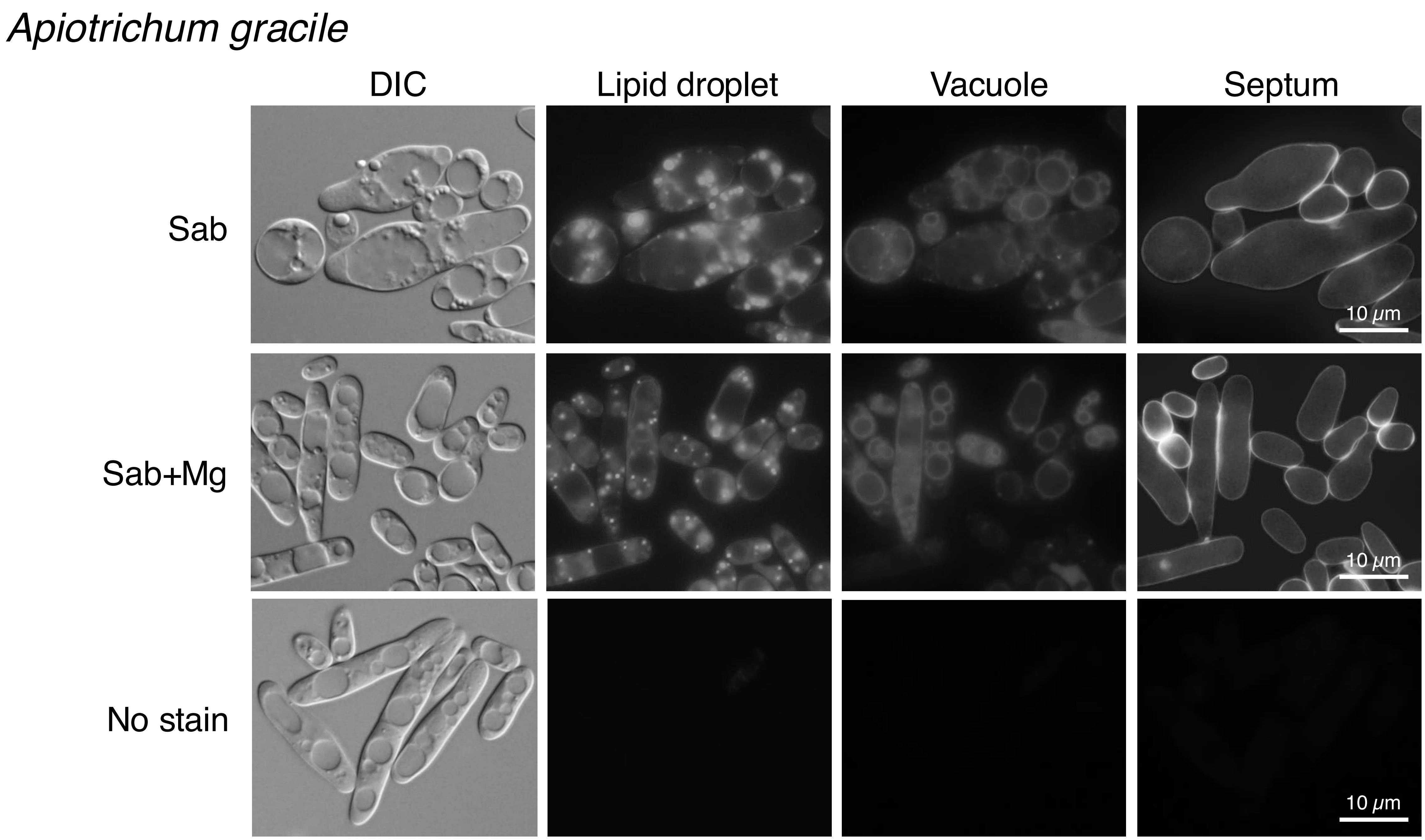

*Apiotrichum gracile*
DIC
Lipid droplet
Vacuole
Septum
Sab
Sab+Mg
No stain
10 μm
10 μm
10 μm

*Apiotrichum veenhuisii*

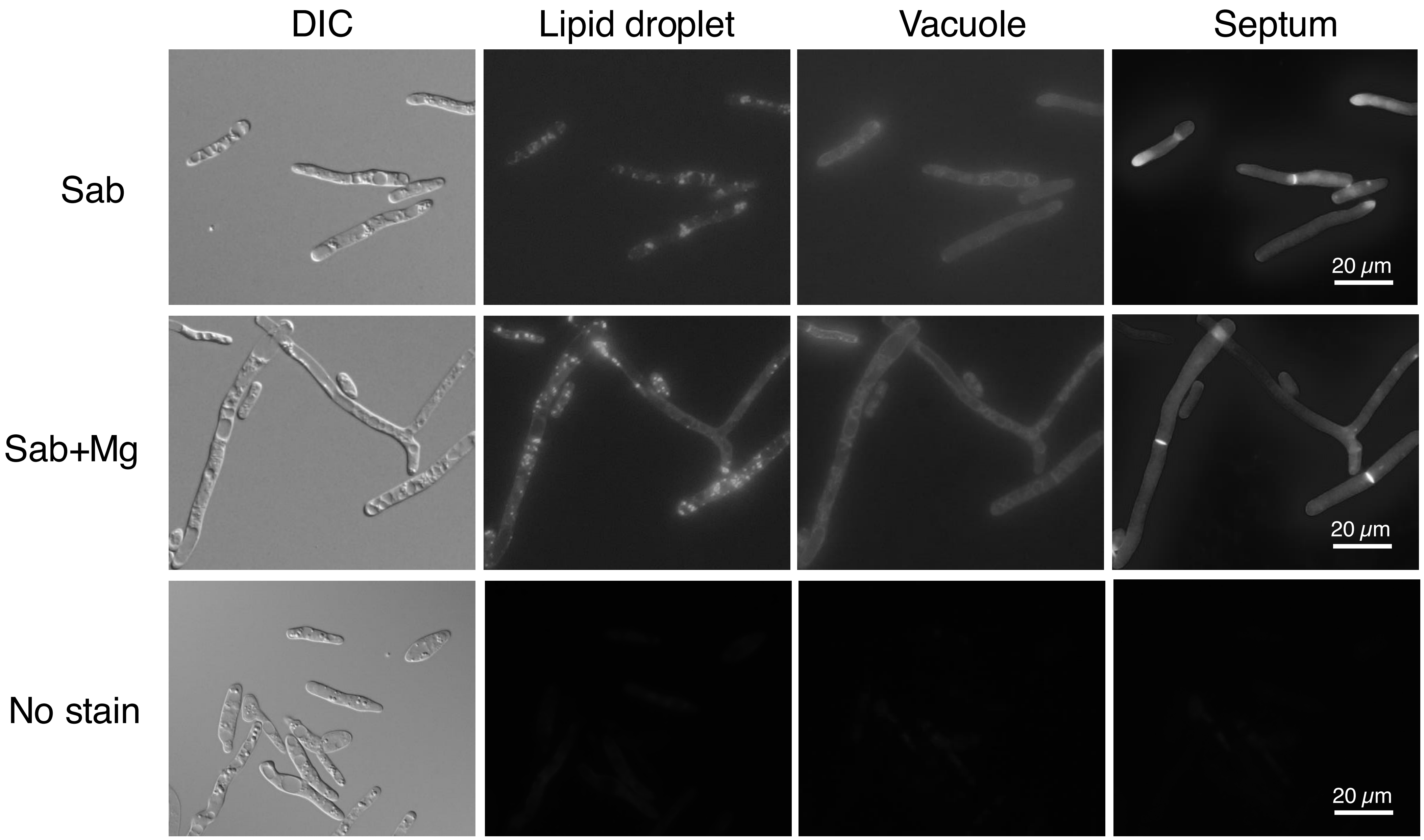

# *Cutaneotrichosporon* genus

*Cutaneotrichosporon spelunceum*

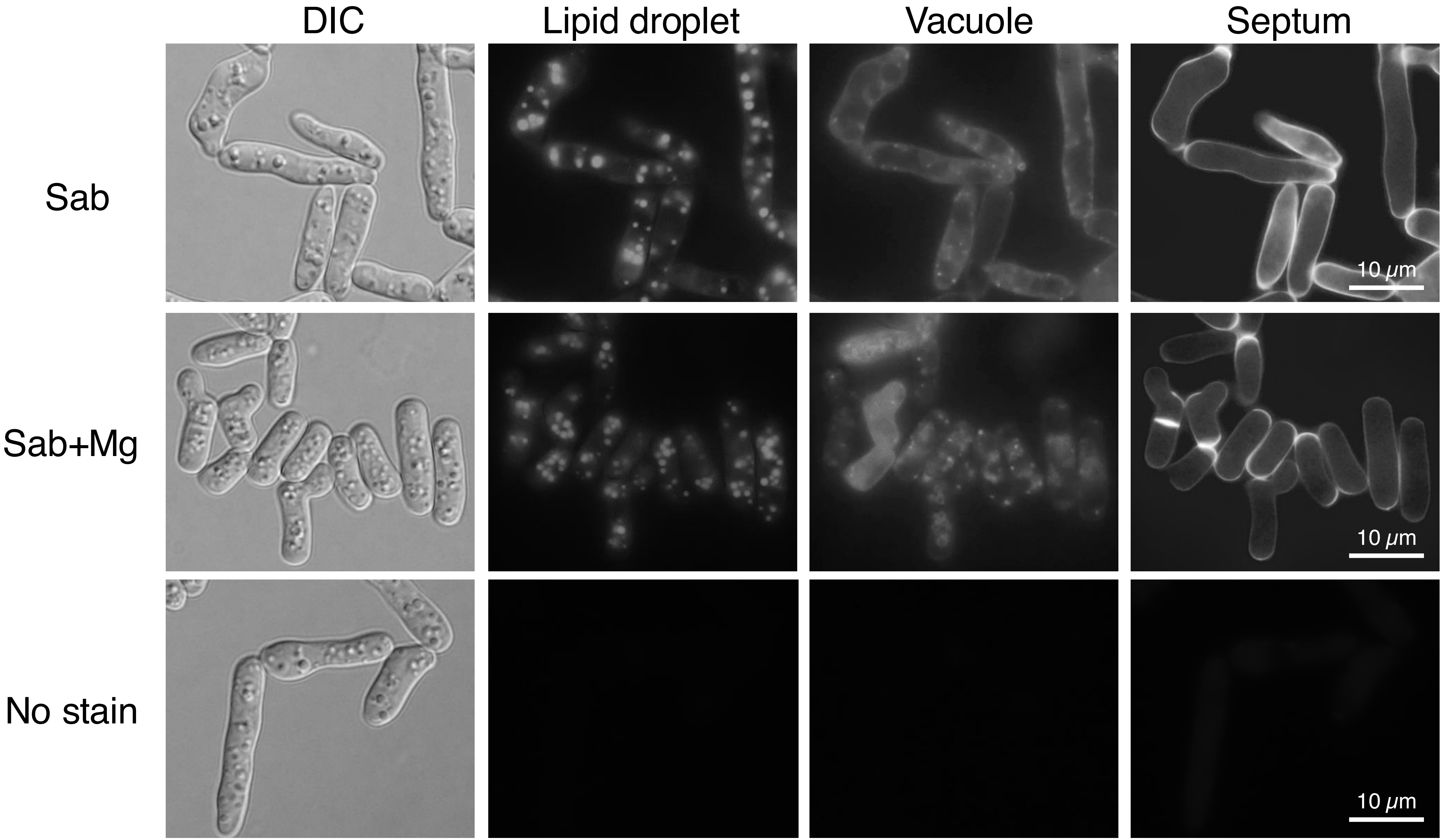

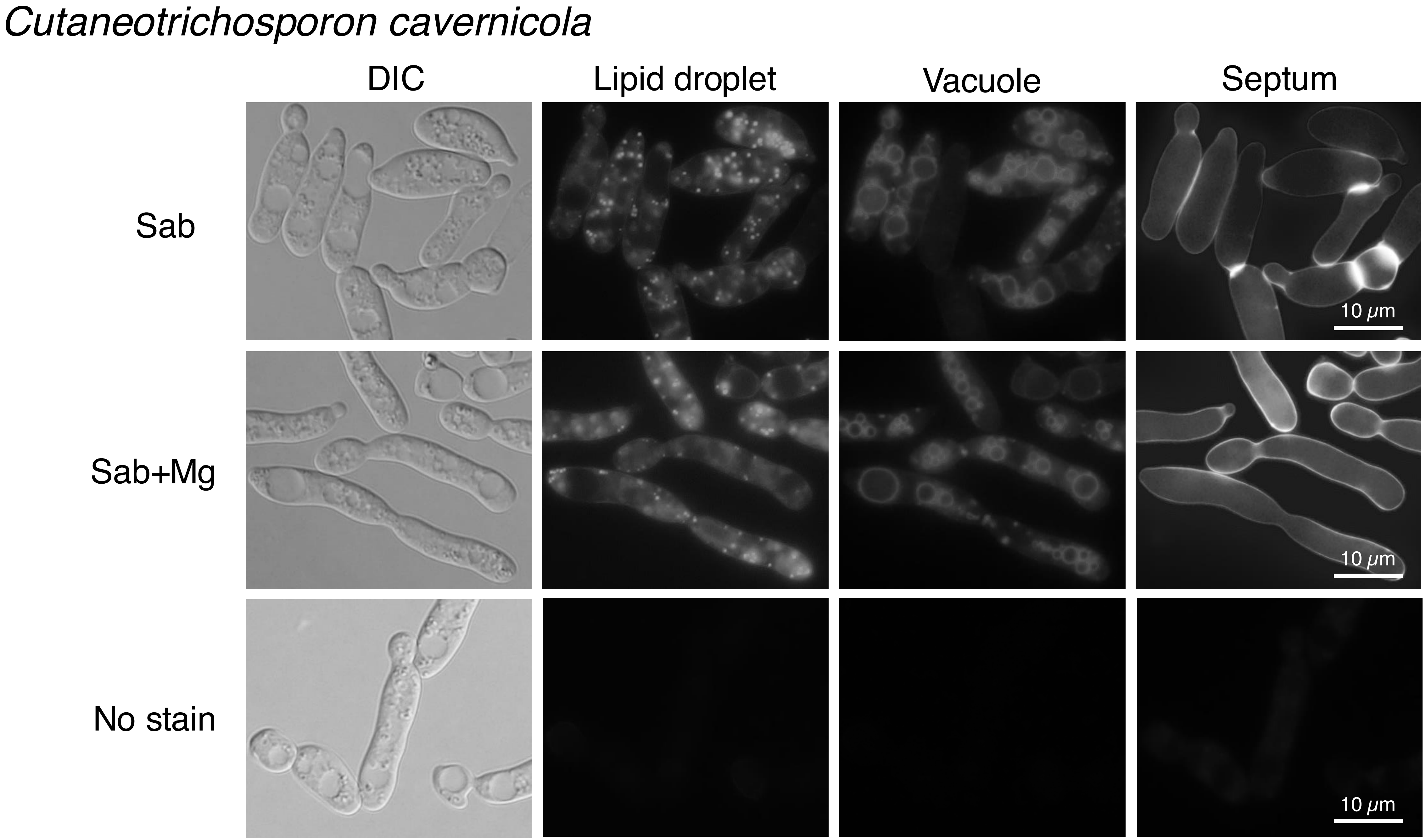

Cutaneotrichosporon cavernicola
DIC
Lipid droplet
Vacuole
Septum
Sab
Sab+Mg
No stain
10 μm
10 μm
10 μm

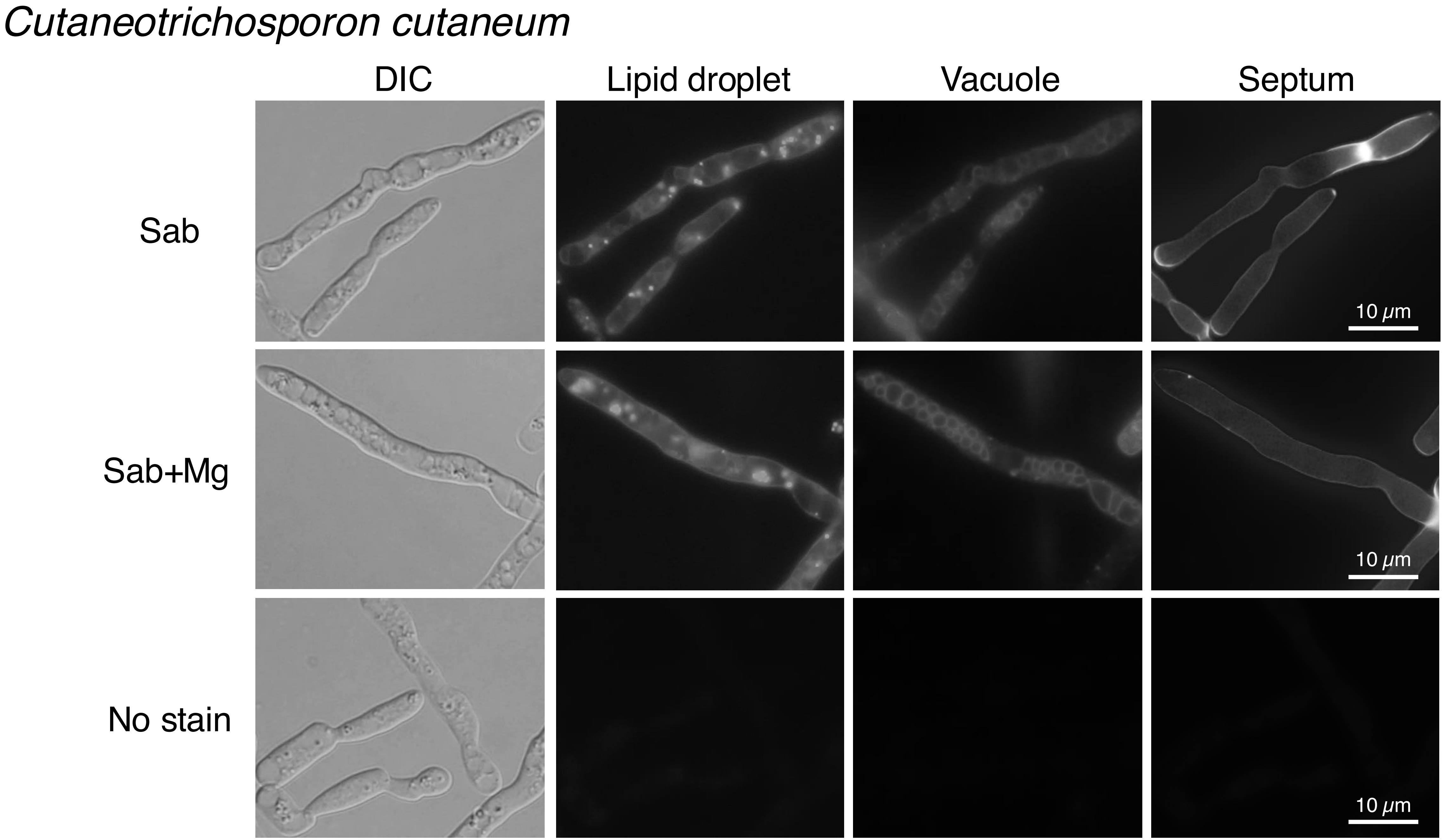
Cutaneotrichosporon cutaneum
DIC
Lipid droplet
Vacuole
Septum
Sab
Sab+Mg
No stain
10 μm
10 μm
10 μm

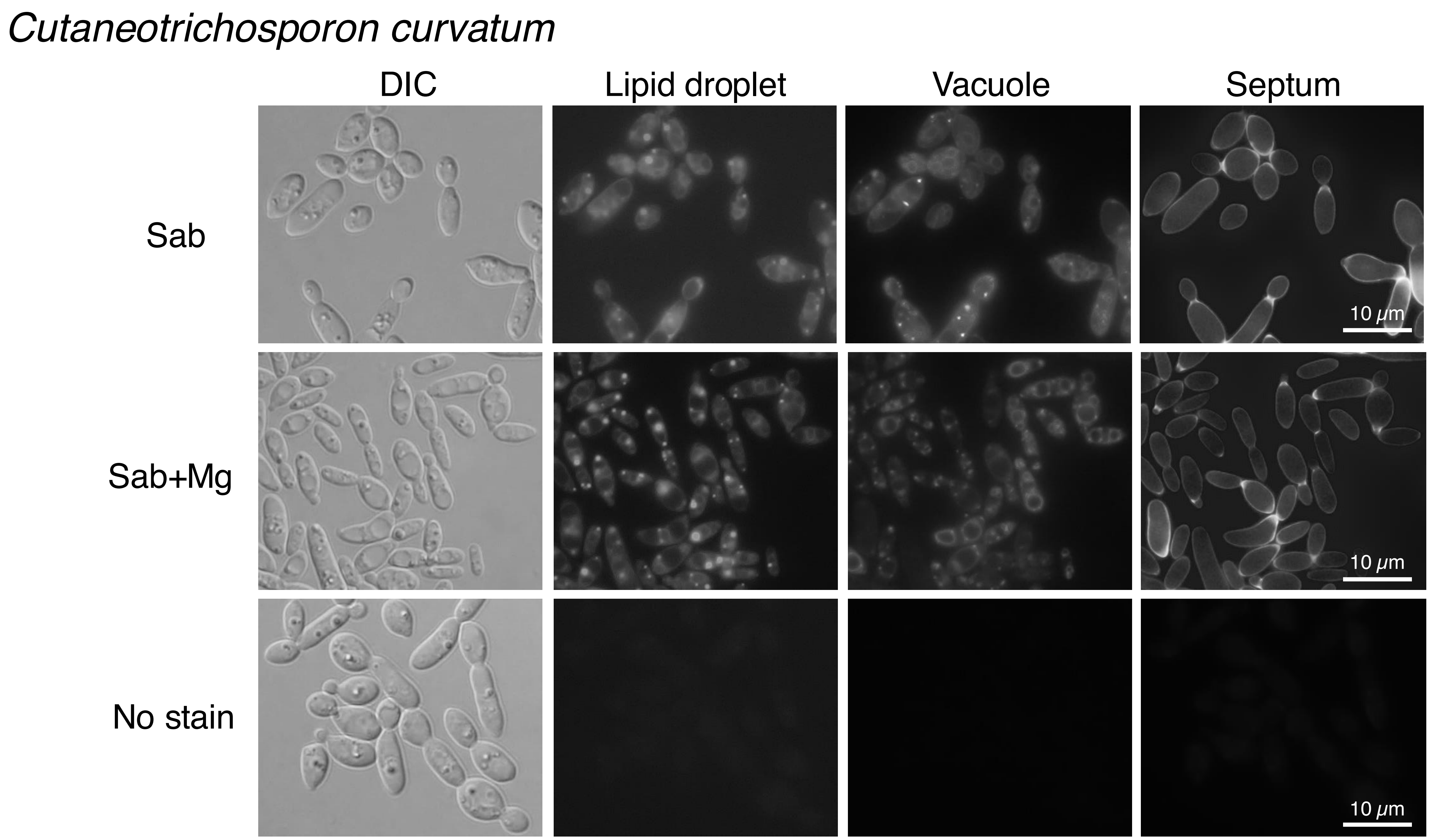

Cutaneotrichosporon curvatum
DIC
Lipid droplet
Vacuole
Septum
Sab
Sab+Mg
No stain
10 μm
10 μm
10 μm

# *Cutaneotrichosporon mucoides*

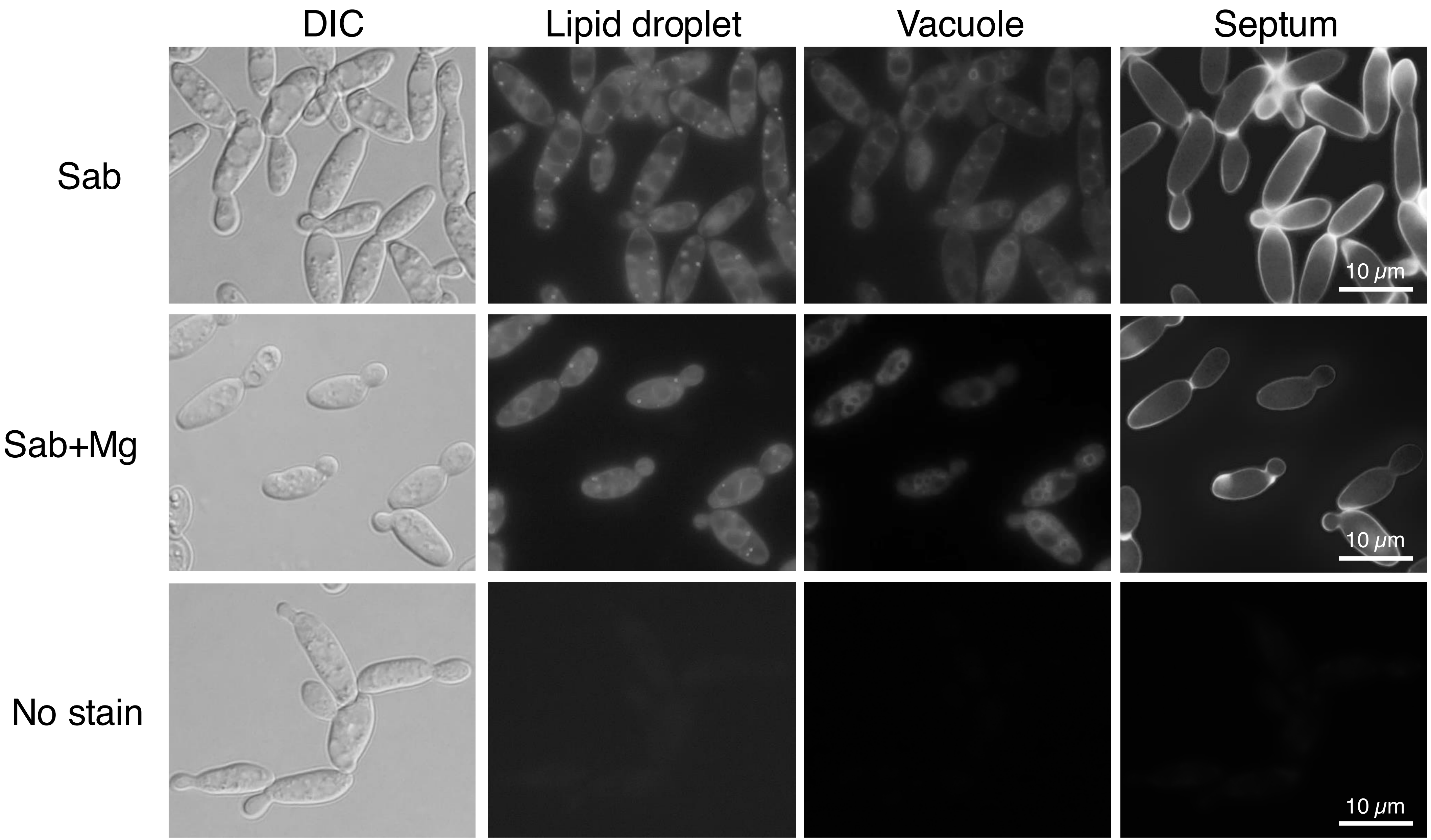

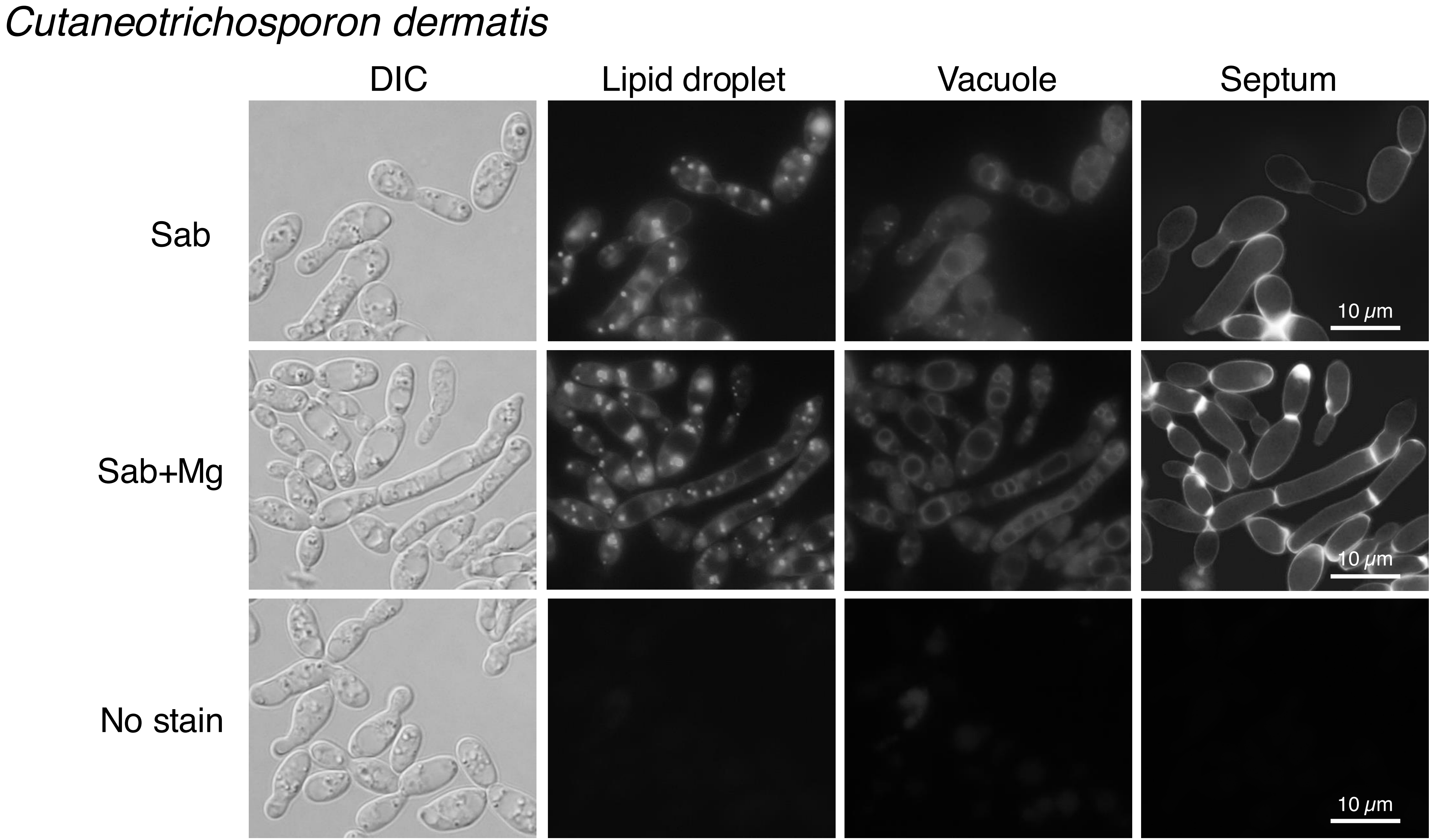

Cutaneotrichosporon dermatis
DIC
Lipid droplet
Vacuole
Septum
Sab
Sab+Mg
No stain
10 μm
10 μm
10 μm

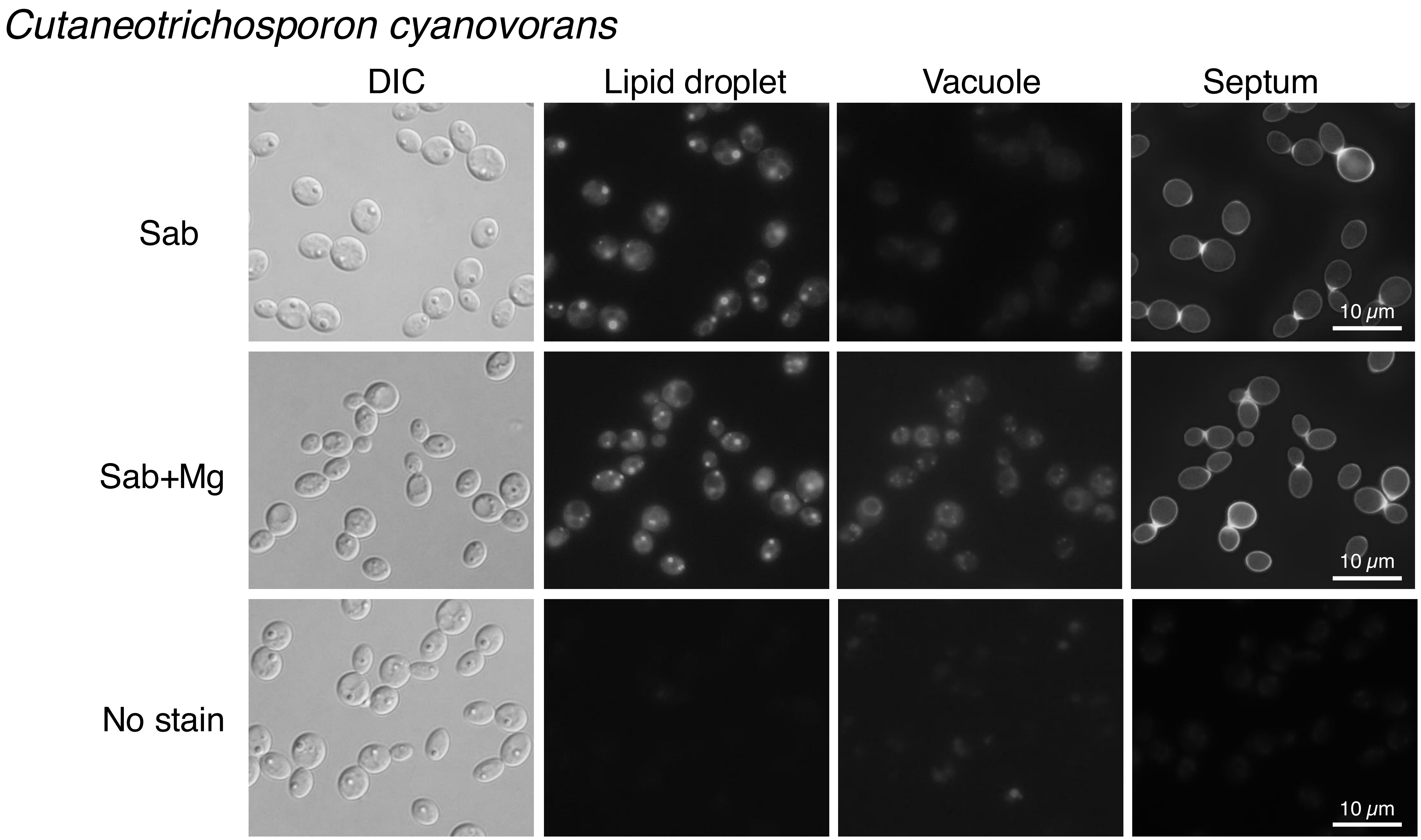
Cutaneotrichosporon cyanovorans
DIC
Lipid droplet
Vacuole
Septum
Sab
Sab+Mg
No stain
10 μm
10 μm
10 μm

*Cutaneotrichosporon daszewskae*

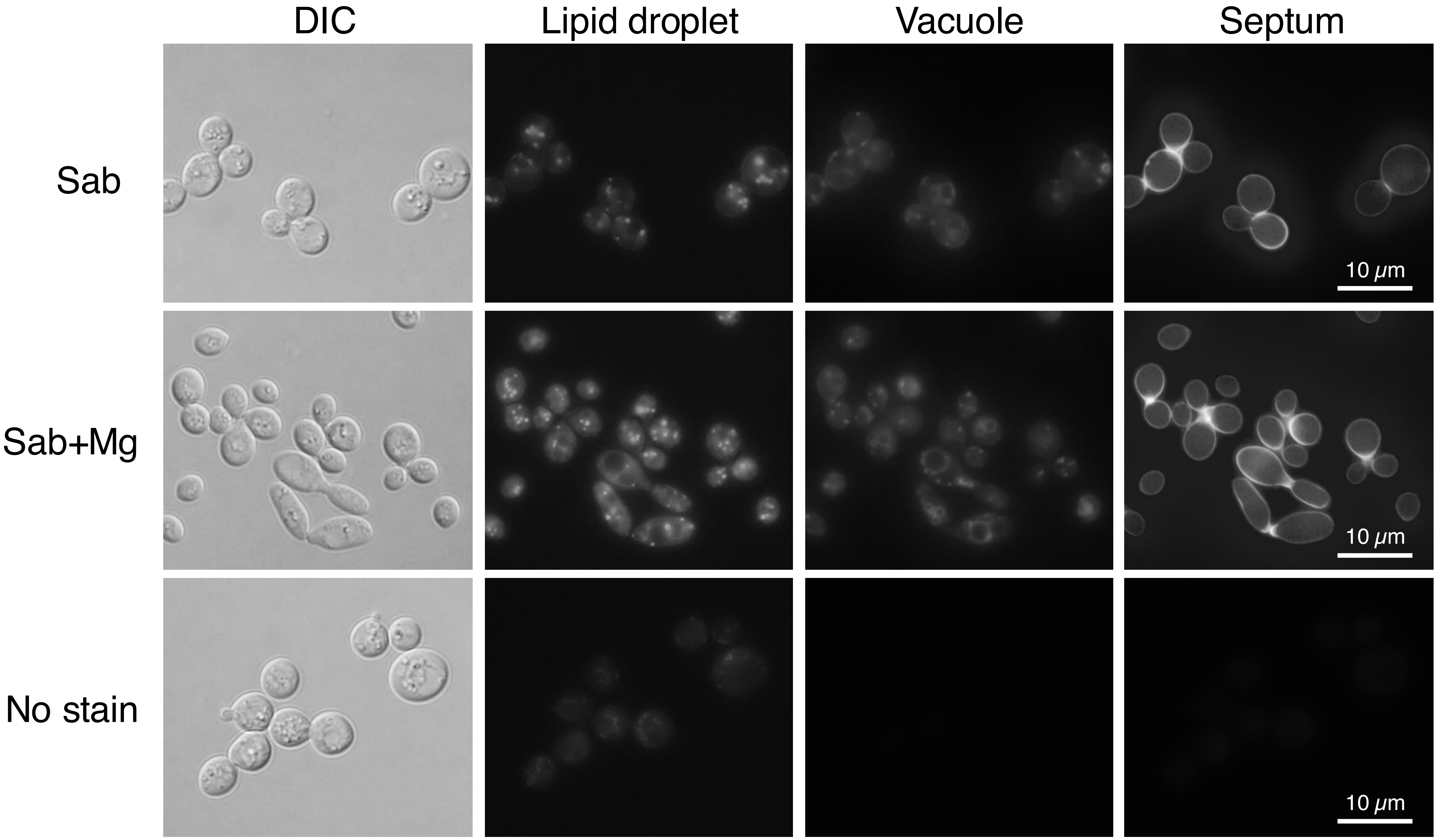

*Cutaneotrichosporon arboriforme*

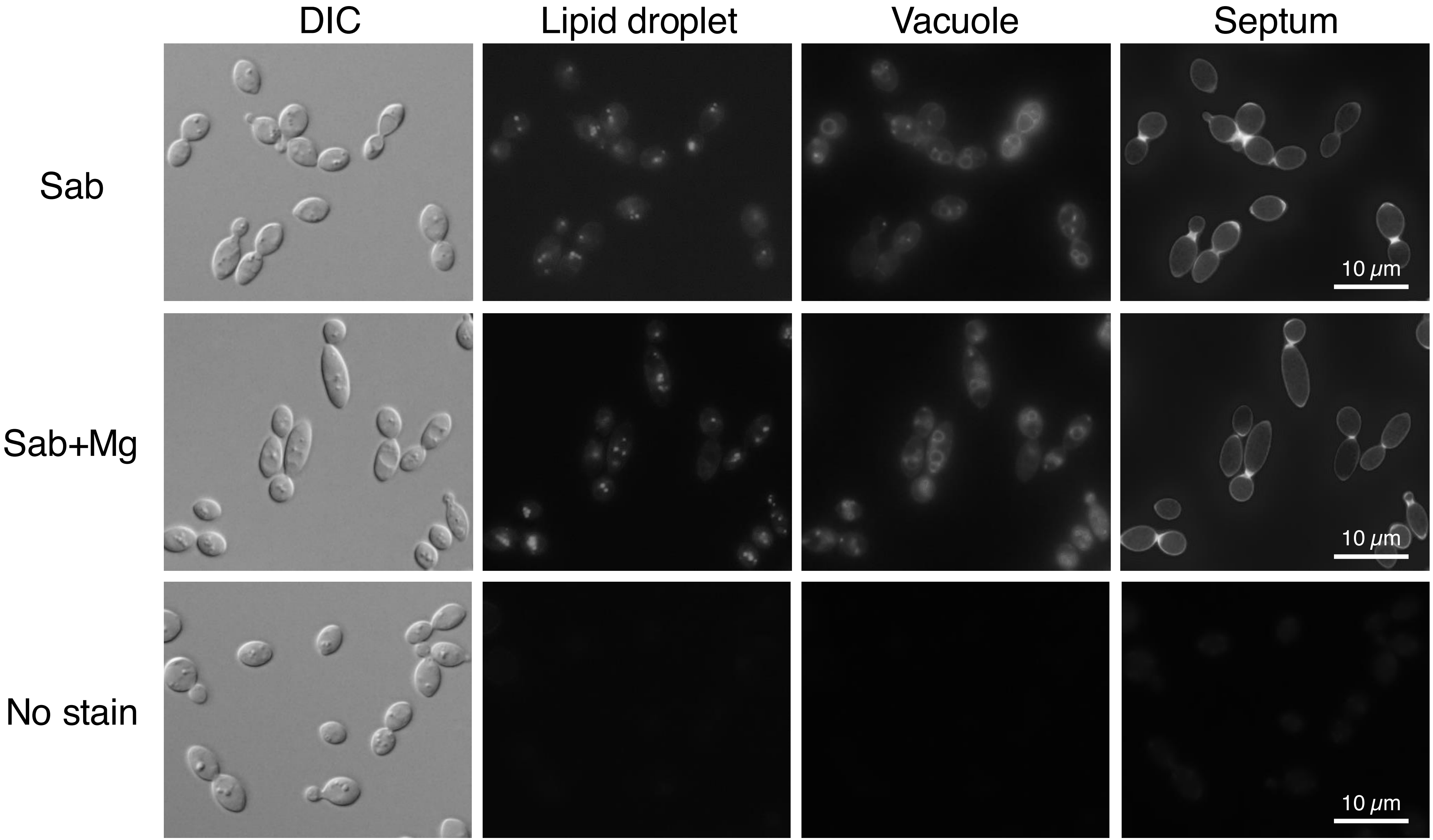

# *Takashimella* genus

# *Takashimella koratensis*

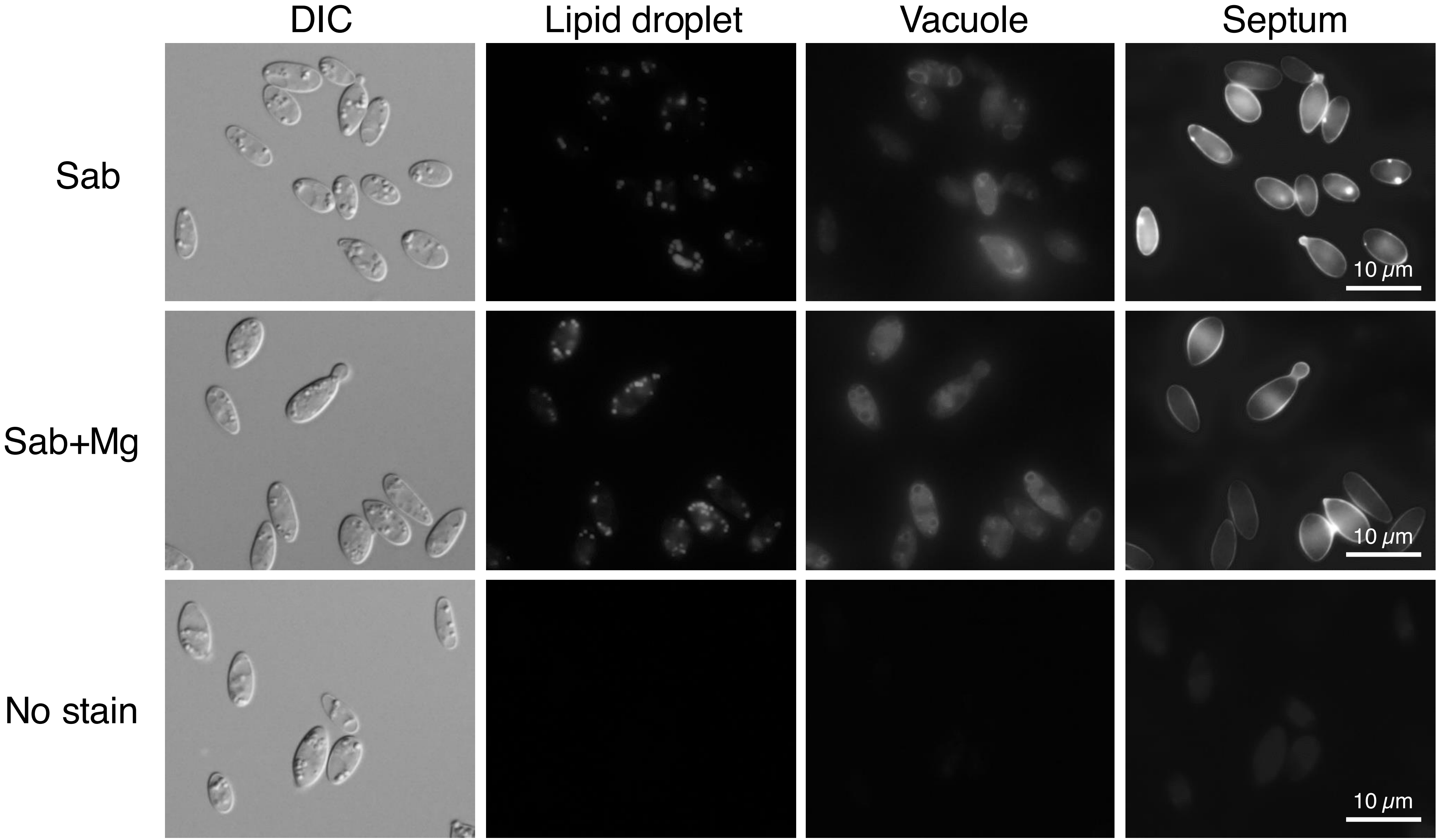

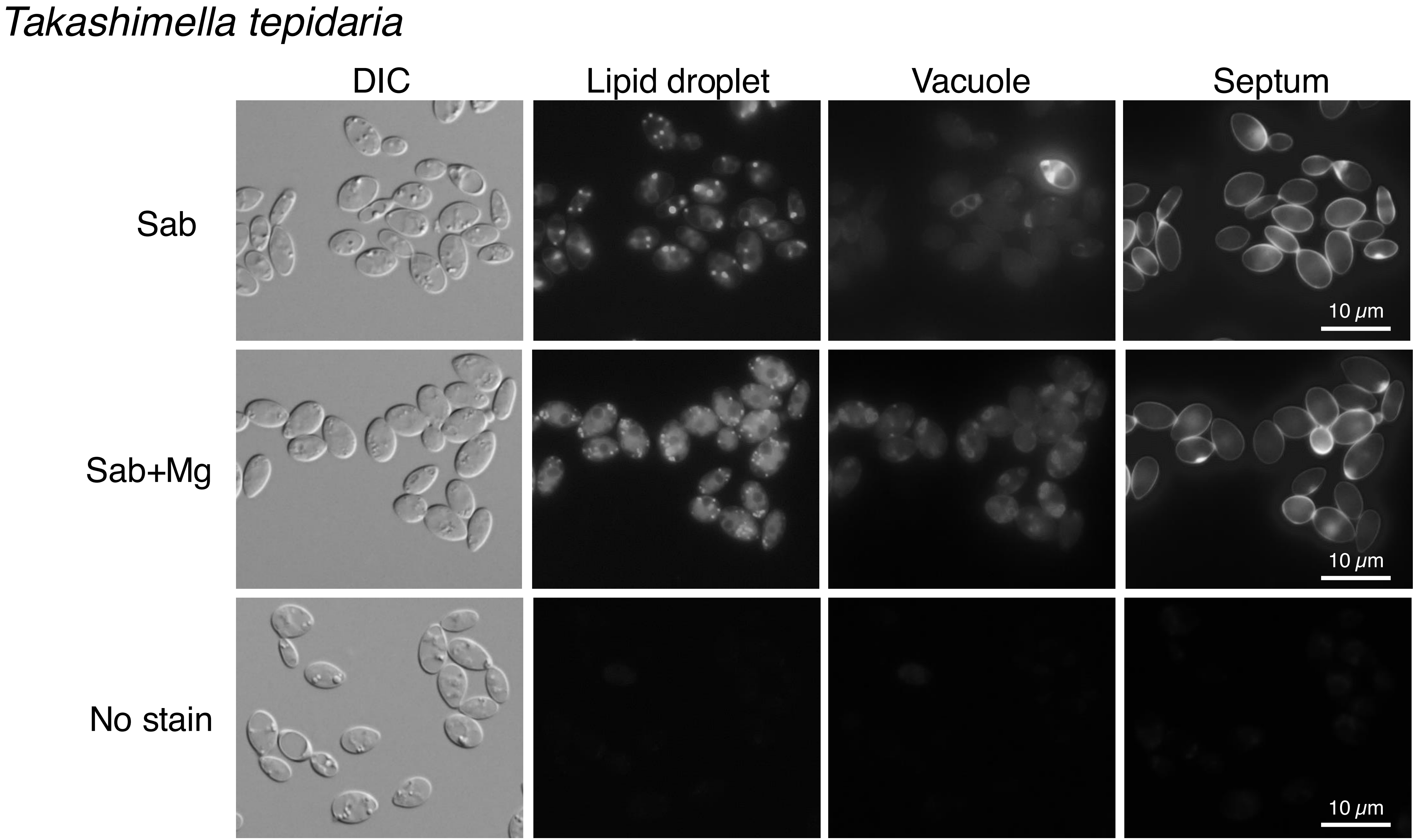

Takashimella tepidaria
DIC
Lipid droplet
Vacuole
Septum
Sab
Sab+Mg
No stain
10 μm
10 μm
10 μm

# Others

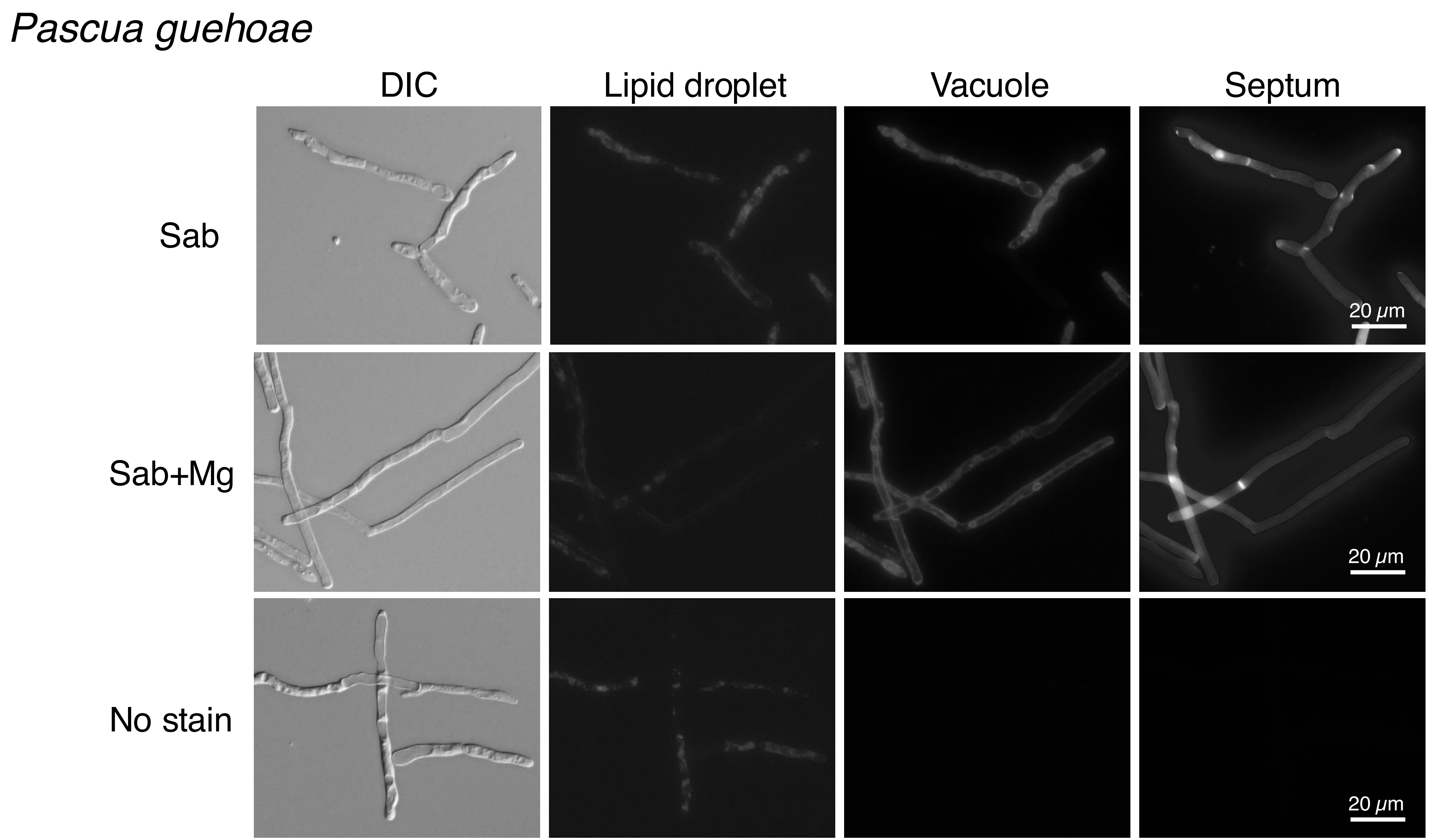

Pascua guehoae
DIC
Lipid droplet
Vacuole
Septum
Sab
Sab+Mg
No stain
20 μm
20 μm
20 μm

# *Prillingera fragicola*

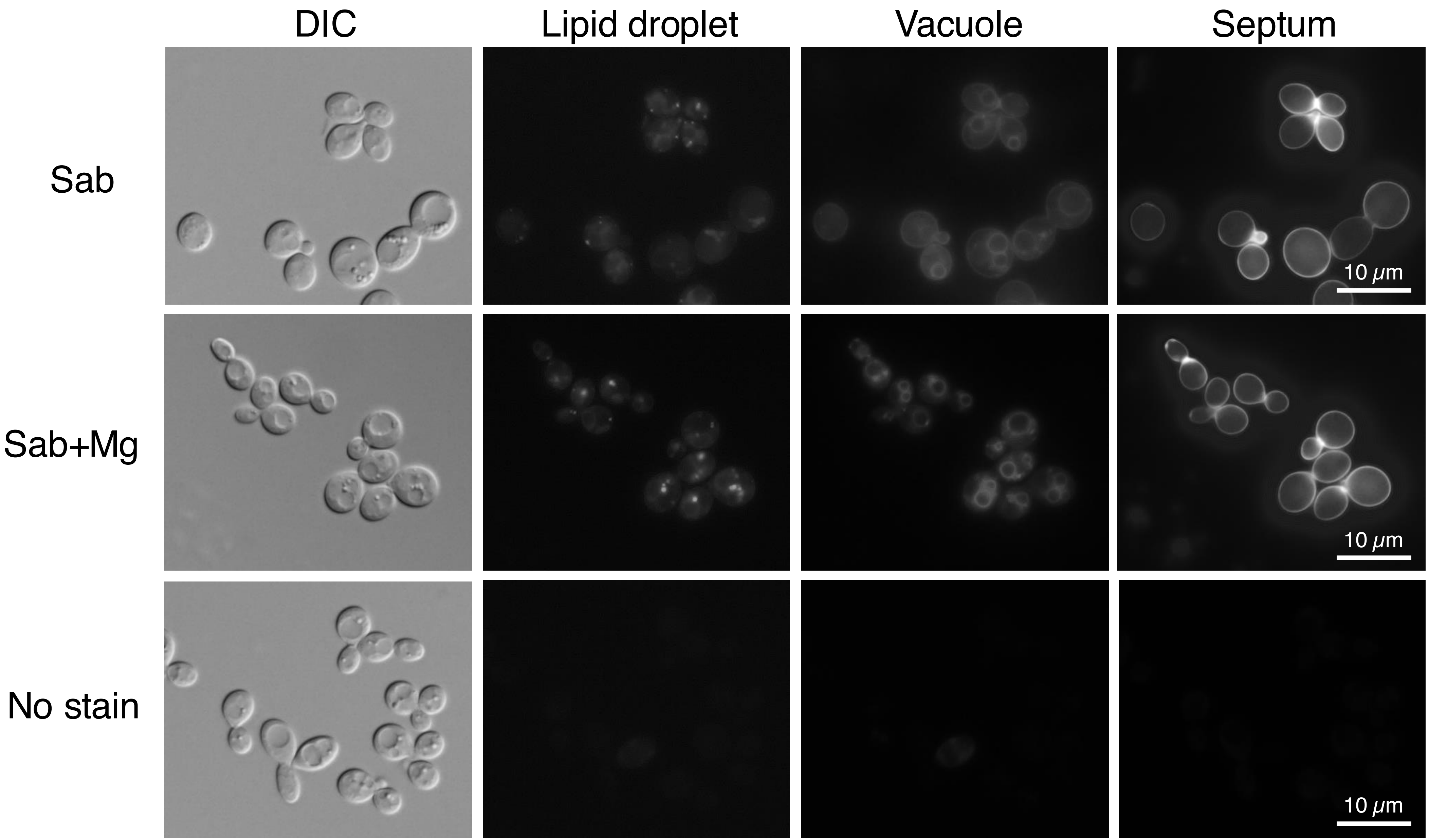

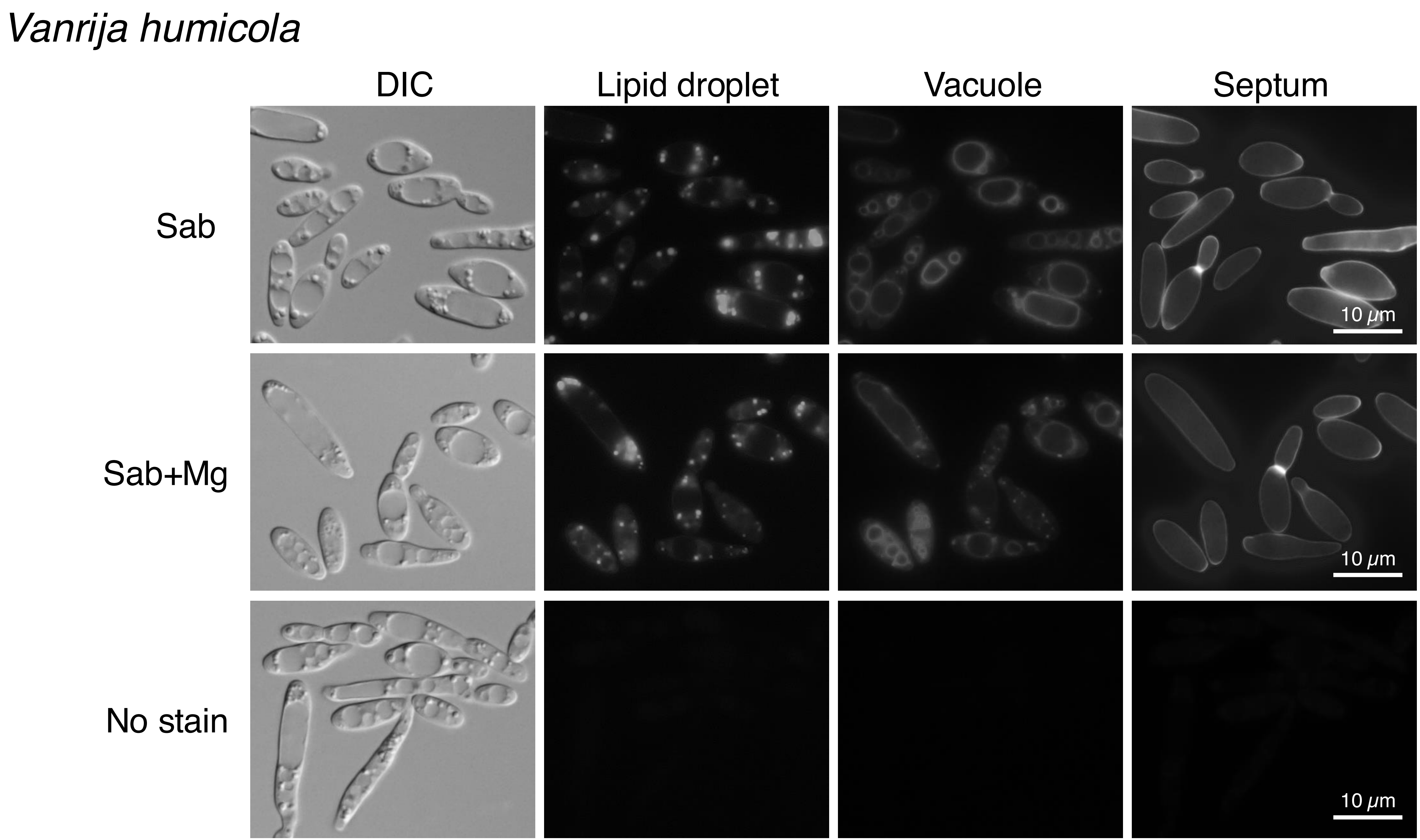
Vanrija humicola
DIC
Lipid droplet
Vacuole
Septum
Sab
Sab+Mg
No stain
10 μm
10 μm
10 μm

# Yeasts out of Trichosporonales

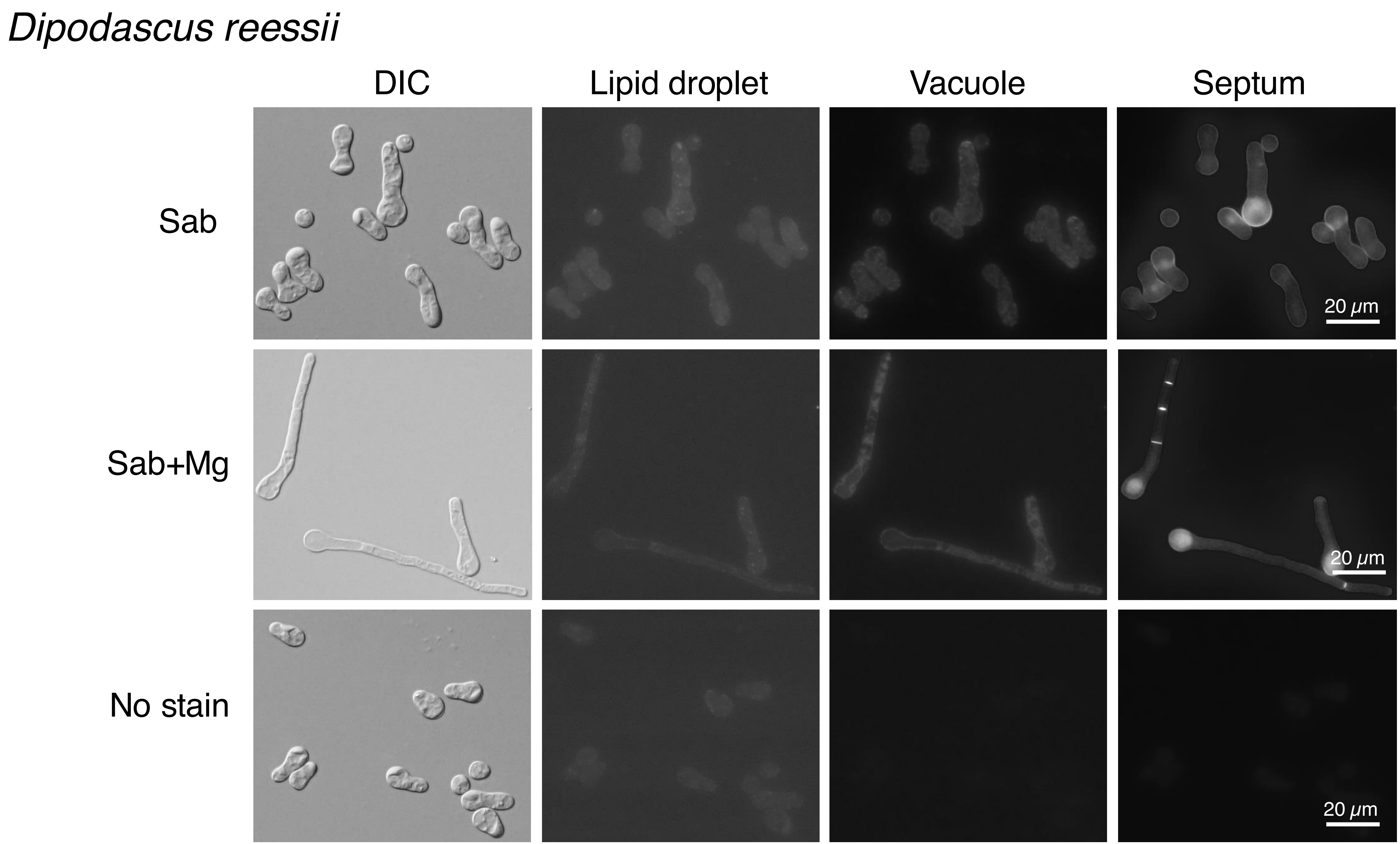
Dipodascus reessii
DIC
Lipid droplet
Vacuole
Septum
Sab
Sab+Mg
No stain
20 μm
20 μm
20 μm

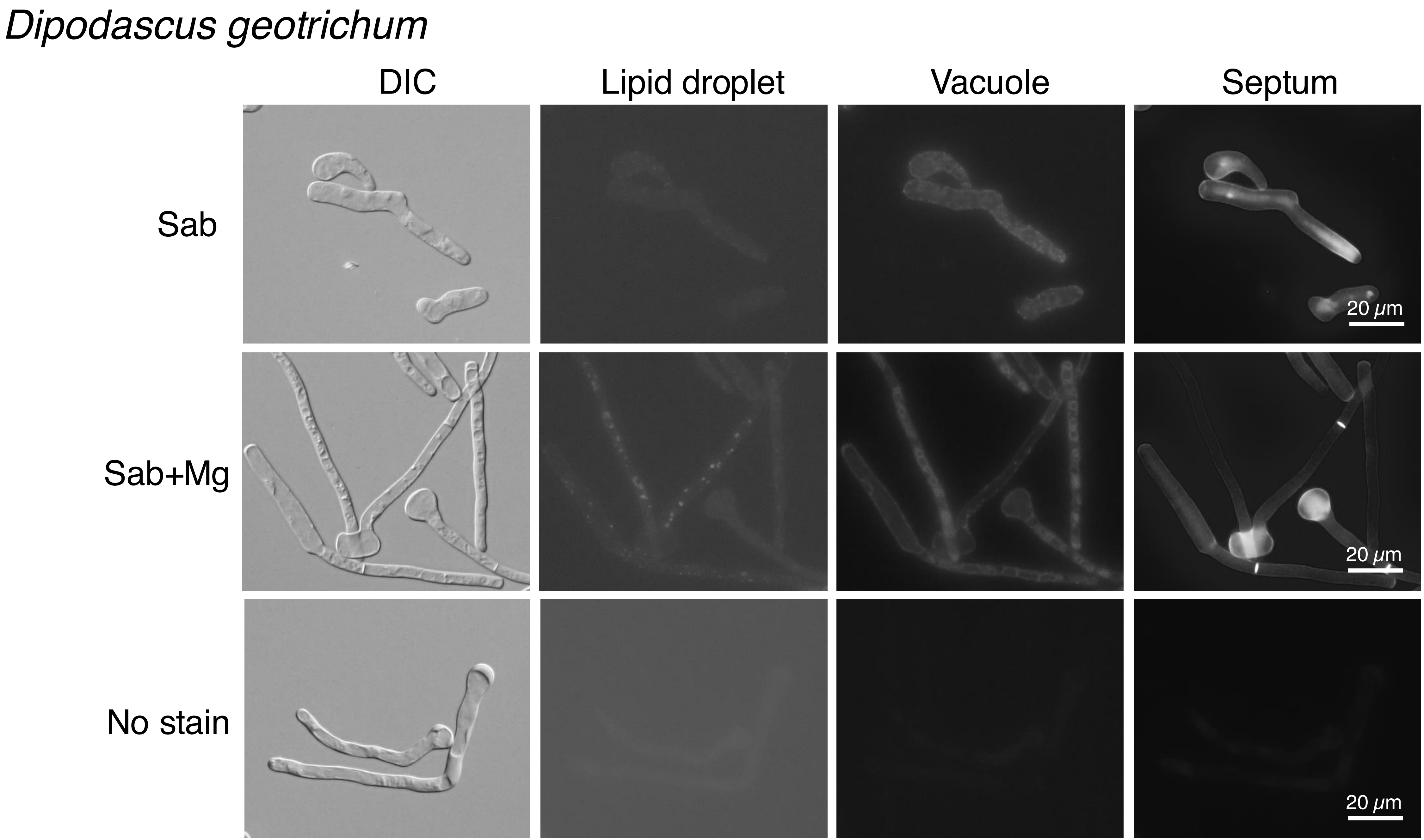

*Dipodascus geotrichum*
DIC
Lipid droplet
Vacuole
Septum
Sab
Sab+Mg
No stain
20 μm
20 μm
20 μm

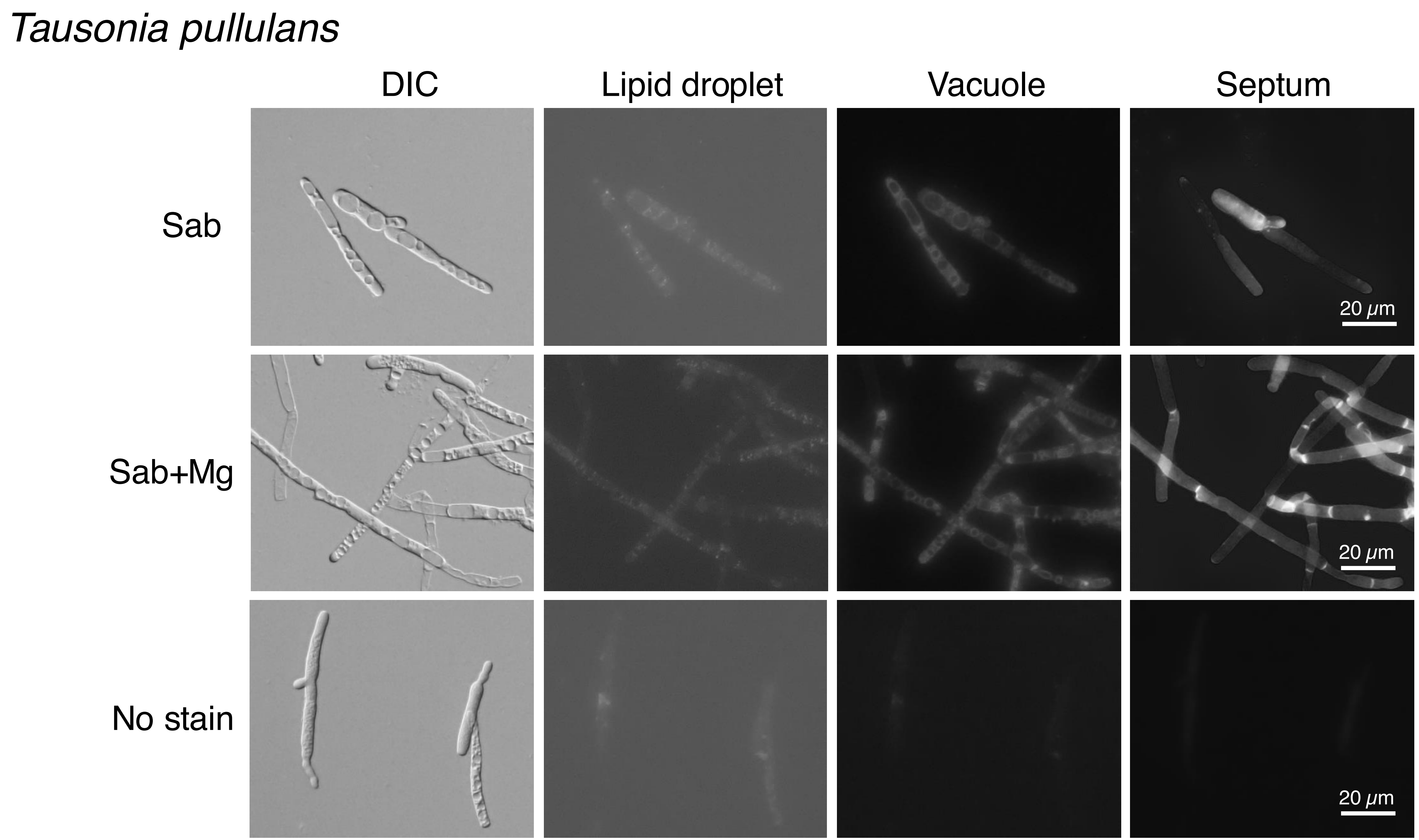

Tausonia pullulans
DIC
Lipid droplet
Vacuole
Septum
Sab
Sab+Mg
No stain
20 μm
20 μm
20 μm

*Schizosaccharomyces pombe*

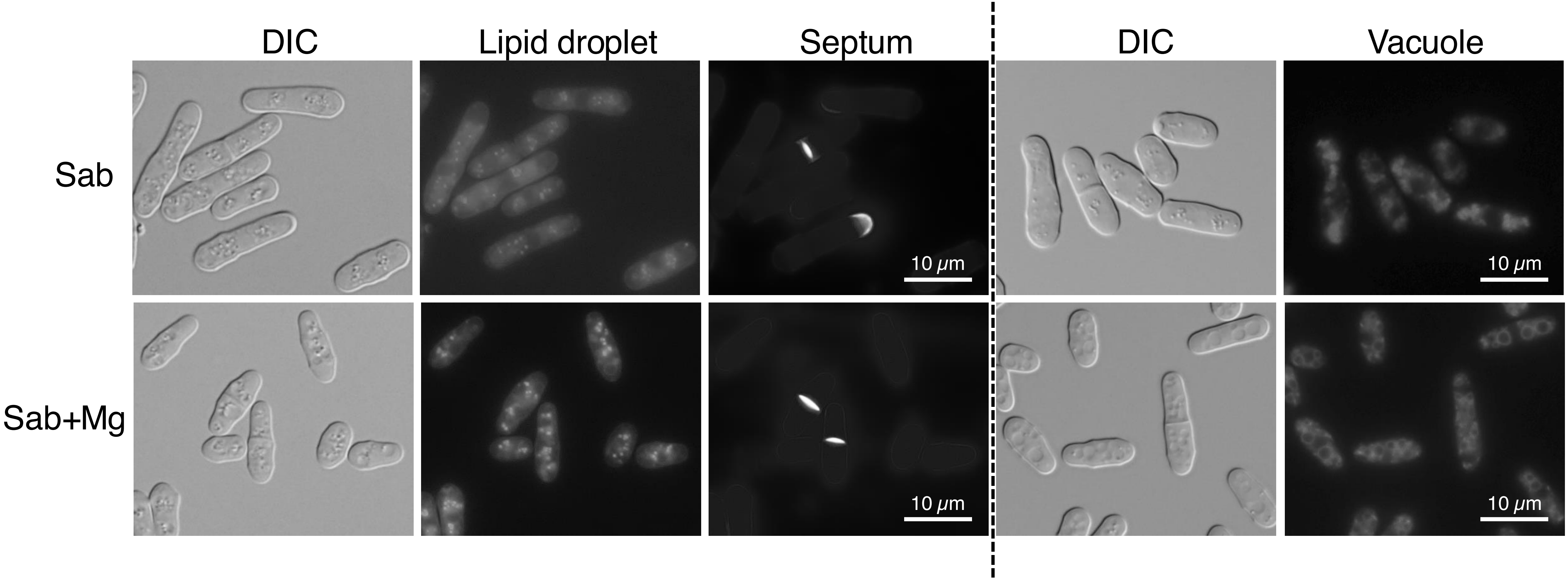

# *Saccharomyces cerevisiae*

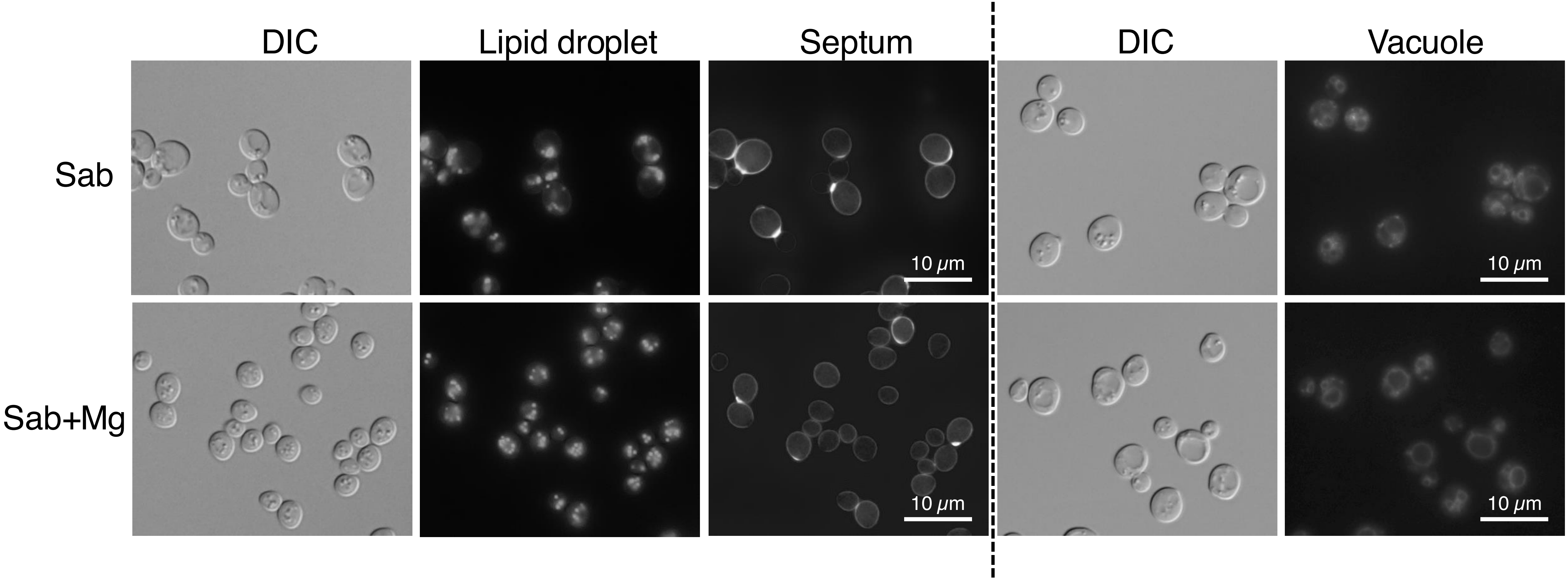

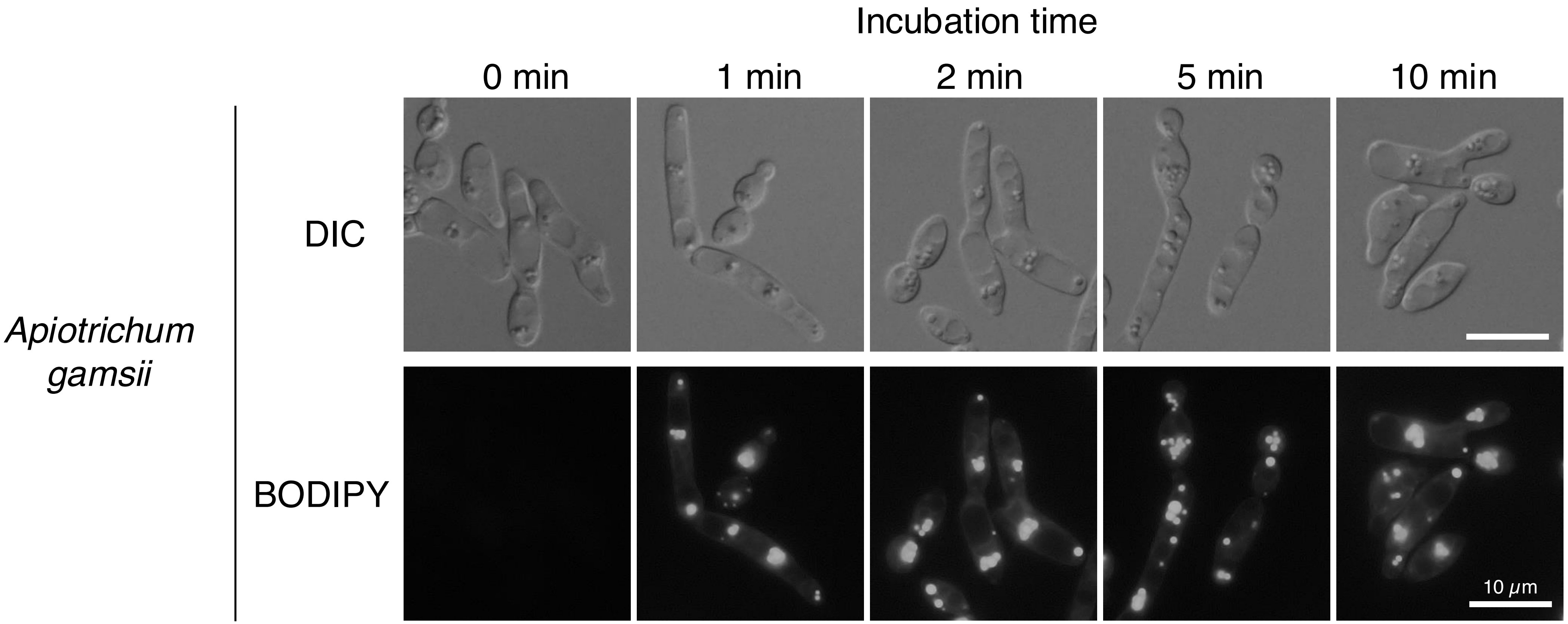


Supplemental Fig. 5

*A. gamsii* cells were cultivated to the density of $OD_{660}$=0.524 in Sabouraud broth supplemented with $MgSO_4$ at 25 ˚C. The cell culture of 5 mL was divided into 5 tubes and washed once with 1 mL PBS, respectively. Thereafter, the cells were mixed with 1 mL PBS containing 100 nM BODIPY, and left in the dark for 0, 1, 2, 5, and 10 min at 25-28 ˚C. The cells were washed twice with 1 mL PBS. They were resuspended in 50 μL PBS before observation. The U-GFP mirror unit was used for observing BODIPY signals. The brightness of BODIPY signals did not depend on the incubation time from 1 to 10 min. The bar is 10 μm.
